# Growth, inequality and poverty: a robust relationship?

**DOI:** 10.1007/s00181-021-02152-x

**Published:** 2021-11-23

**Authors:** Gustavo A. Marrero, Luis Servén

**Affiliations:** 1 Departamento de Economía, Contabilidad y Finanzas, CEDESOG, Universidad de La Laguna, San Cristóbal de La Laguna, Spain; 2 EQUALITAS, Madrid, Spain; 3 CEMFI, Madrid, Spain

**Keywords:** Growth, Inequality, Poverty, Indirect impacts, O40, O11, O15, E25

## Abstract

The consequences of poverty and inequality for growth have long preoccupied academics and policy-makers. This paper revisits the inequality-growth and poverty-growth links. Using a panel of 158 countries between 1960 and 2010, we find that the correlation of growth with poverty is consistently negative: A 10 p.p. decrease in the headcount poverty rate is associated with a subsequent increase in per capita GDP between 0.5 and 1.2% per year. In contrast, the correlation of growth with inequality is empirically fragile—it can be positive or negative, depending on the empirical specification and econometric approach employed. However, the indirect effect of inequality on growth through its correlation with poverty is robustly negative. Closer inspection shows that these results are driven by the sample observations featuring high poverty rates.

## Introduction

What is the effect of poverty on aggregate income growth? And the effect of inequality? Academics and policy-makers have long been concerned with these questions. But they have typically been explored as separate issues. Yet properly answering them requires taking them up jointly, because poverty and inequality are interrelated features of the same income distribution (Bourguignon 2004).

This paper attempts to fill that gap by providing an empirical exploration of the growth effects of both poverty and inequality and, in particular, of their respective robustness. The effects of poverty have been analyzed by numerous theoretical papers highlighting a variety of mechanisms through which poverty may become self-perpetuating. But empirical work has been more limited and largely inconclusive. Indeed, a basic implication of the theoretical models of poverty traps—namely, that countries suffering from higher levels of poverty should grow less rapidly than comparable countries with lower poverty—has been largely overlooked. This is the key hypothesis pursued in this paper. It can be viewed as a weak version of the poverty trap hypothesis, in that to support it we do not need to find evidence of multiple equilibria or income stagnation, but just empirical proof that, other things equal, poverty tends to hold back growth.

In contrast, the effects of inequality have attracted massive empirical literature, albeit with sharply conflicting results. The present paper adds to existing work by highlighting a novel angle, namely the indirect effect of inequality on growth accruing through the impact of inequality on poverty: given the poverty line and the overall population’s mean income, an increase in inequality will typically raise poverty, by pushing more individuals below the poverty line.^1^ If poverty affects growth, so will inequality through this indirect channel—in addition to any direct effects that inequality might exert on growth.

To assess the respective growth impacts of poverty and inequality, we estimate a reduced-form growth equation with inequality and poverty added separately and jointly to an otherwise standard set of growth determinants (educational attainment, investment prices, government size, degree of openness, public infrastructures, etc.). For the estimation, we assemble a large panel data set of non-overlapping five-year observations comprising 158 countries over the period 1960–2010. The sample is heavily unbalanced, and its size exceeds by far that found in earlier studies of the poverty-growth link.

Our econometric approach is based on GMM estimation employing internal instruments (Arellano and Bover 1995; Blundell and Bond 1998; Roodman 2009). In our setting, the choice of this approach is dictated by the short time dimension and large cross-sectional dimension of our panel dataset—which makes panel time-series methods unsuitable—and by the potential endogeneity of the regressors—which demands an instrumental variable approach. These issues affect also much of the empirical literature on the links between poverty, inequality and growth, which—like our paper—has to contend with the potential problem of two-way causality between the variables at the core of the analysis.

In this context, GMM represents a natural methodological choice, which we also share with much of the related empirical literature.^2^ Moreover, this common empirical methodology also makes our paper more easily comparable with existing work. Finally, while our use of GMM for growth empirics is not novel, our paper is among the first to examine rigorously, in a system GMM setting, the potential problem of weak instruments plaguing much of the empirical growth literature, as first raised by Kraay (2015) in the context of the empirical relationship between inequality and growth.

Our main finding is that poverty has a robust negative and significant effect on growth. As for inequality, we find that the sign and significance of its direct effect on growth are fragile. However, its indirect effect (through poverty) is robustly negative. Further inspection reveals the presence of nonlinearities, in that these results are driven by the sample observations featuring high poverty: when poverty is low, its impact on growth is not significant, and the indirect effect of inequality on growth is therefore absent. We reach a similar conclusion when we let the growth impact of poverty differ between developed and developing countries: It is negative and significant for the latter, but not for the former.

Our results survive a battery of robustness checks, including the use of alternative sets of instruments and specifications in the GMM estimation, different poverty lines and poverty measures, alternative poverty data, nonlinear and nonparametric specifications, or the use of alternative sets of control variables. We also find that our preferred GMM specification can address in a satisfactory manner the endogeneity, under-identification and weak instruments problems often encountered in macroeconomic applications of dynamic panel models (Bazzi and Clemens 2013).

Our paper is embedded in an extensive literature (recently surveyed by Cerra et al. 2021a) analyzing the multidirectional links among growth, inequality and poverty. Three strands are especially relevant in our context. They, respectively, focus on the impact of poverty on growth, the impact of inequality on growth, and the contribution of inequality and income growth to poverty. We provide a brief review of these literature works in the next section.

The rest of the paper is structured as follows. As just noted, Sect. 2 is devoted to a selective summary of the literature on the growth-inequality-poverty nexus. In Sect. 3, we describe the data and we lay out the empirical strategy to test for the effects of poverty and inequality on growth. In Sect. 4, we report the main empirical results for our baseline specification. Section 5 reports extensive robustness checks on our empirical results. Section 6 analyzes how the links of poverty and inequality with growth might depend on the prevailing degrees of poverty and/or inequality and gauges the direct and indirect effects of inequality on growth. Finally, Sect. 7 concludes.

## The growth-inequality-poverty nexus: a review

The seminal work of Kuznets (1955) is the starting point of an extensive literature analyzing the growth-inequality-poverty nexus (see Bourguignon 2004, and the recent surveys by Cerra et al. 2021a, b). Our paper relates to several strands of this literature.

First, a long-standing theoretical literature has studied a variety of mechanisms through which poverty may deter economic growth. Its arguments are mostly based on the existence of poverty traps, i.e., mechanisms through which poverty prevents a significant share of the population from helping ignite the growth engine (Azariadis and Stachurski 2005; Bowles et al. 2006; Haider et al. 2018). Under appropriate conditions, those mechanisms may lead to multiple equilibria and make the negative impact of poverty on growth self-reinforcing. In general, the mechanisms highlighted in the literature operate by reducing the incentives and/or abilities of the poor to undertake risky entrepreneurial activities, and/or to accumulate physical and human capital.

A prominent mechanism involves ‘threshold effects’ (Azariadis and Drazen 1990), resulting, for example, from indivisibilities or increasing returns to scale.^3^ For example, if poverty is coupled with credit constraints, the result is that below a certain level of income or wealth economic agents may be too poor to afford the investments (in human or physical capital) or the technologies necessary to raise their income (Galor and Zeira 1993; Banerjee and Newman 1993). Malnutrition provides another example. In developing countries, poverty is associated with high rates of malnutrition (Dasgupta and Ray 1986), which impacts cognitive abilities and school absenteeism and is transmitted to the children’s capacity to learn. The resulting educational inequality is also growth-deterring (Galor and Moav 2004).

Institutional arrangements that place economic opportunities beyond the reach of the poor can likewise result in reduced income growth (Mookherjee and Ray 2002; Engerman and Sokoloff 2006). Another poverty-perpetuating mechanism is related to risk aversion (Banerjee 2000): Because poorer individuals are typically more risk averse, in the absence of well-functioning insurance and credit markets, they will skip profitable investment opportunities that they deem too risky.^4^ Poverty can also alter the decision-making process of individuals toward less growth-enhancing activities. For instance, the poor devote a significant fraction of their income to satisfying basic needs (Shah et al. 2012) and to “temptation” goods (Banerjee and Mullainathan 2010) and reduce the resources devoted to education, health and investment. Poor individuals show also lower aspirations, as they anticipate that their current status will impede their future success (La Ferrara 2019).

In spite of the diversity of these analytical models, evidence on their empirical relevance remains largely inconclusive. A few papers (see Durlauf 2006, for a review) have searched for various empirical regularities consistent with those models, such as aggregate non-convexities (Azariadis and Stachurski 2005) and convergence clubs (Quah 1993). A broader empirical review of different mechanisms advanced in the literature finds little evidence that they may be at work, except perhaps in remote or disadvantaged areas (Kraay and McKenzie 2014). More recently, large-scale randomized evaluations, such as the one developed by Bandiera et al. (2017) in Bangladesh, yield strong evidence that the poor face imperfections in capital markets that keep them in a low asset-low employment poverty trap.

Somewhat surprisingly, just a few papers have taken up the fundamental aggregate implication of the poverty trap literature—that, *ceteris paribus,* countries with higher poverty should grow more slowly. The list is limited to our working paper version, Marrero and Servén (2018), plus López and Servén (2015) and Ravallion (2012), all of which conclude that poverty is growth-deterring^5^; Easterly (2006) shows a non-significant impact of poverty on growth.

The second strand of literature to which our paper is related is concerned with the impact of inequality on growth. It includes a large number of empirical contributions reaching conflicting conclusions; for overviews, see Voitchovsky (2011), Berg et al. (2018), and Cerra et al. (2021a). For example, Alesina and Rodrik (1994) and Perotti (1996) found a negative relationship between inequality and growth in cross section data, but subsequently, Li and Zou (1998) and Forbes (2000) obtained the opposite result using panel data. Barro (2000) found that inequality might affect growth in different directions depending on the country’s level of income, while Panizza (2002) found that results might depend on the model specification and the quality and type of data (see also Deininger and Squire 1998). In turn, Banerjee and Duflo (2003) concluded that the response of growth to inequality changes has an inverted U-shape.

The multiplicity of factors affecting both inequality and growth might explains these contradictory results. For example, rising inequality could be the result of growth-enhancing technological change whose returns are captured by talented individuals at the top of the distribution (Goldin and Katz 2008). In contrast, if rent-seeking is the fundamental force behind growing incomes of the rich, the increase in inequality could come along with declining growth (Stiglitz 2012).

In this line of enquiry, Galor and Moav (2004) argue that the replacement of physical capital accumulation by human capital accumulation as a prime engine of economic growth has changed the qualitative impact of inequality on growth. Marrero and Rodríguez (2013) emphasize that the sign of the effect of inequality on growth depends on the type of inequality considered (i.e., inequality of opportunity or of effort). Voitchovsky (2005) and, more recently, van der Weide and Milanovic (2018) argue that the effect of inequality is negative for the income growth of the poor but positive for the income growth of the rich—i.e., inequality tends to be self-reinforcing. The effects of inequality on growth might also depend on the sectoral structure of the economy (Erman and te Kaat 2019) and on the degree of intergenerational mobility (Aiyar and Ebeke 2020).^6^

In general, different mechanisms affecting growth in opposite directions through different channels act all simultaneously, leading to conflicting inferences. In the empirical literature, an emerging consensus view is that the long-run effect of inequality on growth is significantly negative, and only when looking at relatively short periods of time, the relationship may turn positive (Halter et al. 2014; Brueckner et al. 2015; Berg et al. 2018; Brueckner and Lederman 2018).^7^

A third strand of the literature explores the links between growth and inequality, on the one hand, and poverty, on the other. The bulk of this literature, which is quite extensive (Cerra et al. 2021a), focuses on the poverty-reducing effect of growth and the factors that shape it (Dollar and Kraay 2002; Bourguignon 2003; Ravallion 2004). This angle of the poverty-growth link is the opposite to that pursued in this paper.

Empirically, there is ample consensus that growth reduces poverty—i.e., it is “good for the poor.” Dollar and Kraay (2002), and the subsequent updates using alternative databases and empirical approaches (Kraay 2006, Dollar et al. 2016) find that the income of the poorest deciles varies in the same proportion as average income, hence fostering aggregate growth is pro-poor (see also Ferreira et al. 2010, or Loayza and Raddatz 2010). Recent work confirms this result (Fosu 2017; Bluhm et al. 2018; Bergstrom 2020). For example, Bergstrom (2020) finds that, in a large cross-country sample, 90% of the variation in poverty is explained by variation in per capita GDP. However, the reason is that the sample variation in per capita income is much larger than that of inequality; indeed, in most of the sample countries, the estimated inequality elasticity of poverty exceeds the income elasticity of poverty—which suggests that declines in inequality offer a large potential (as yet unrealized) to reduce poverty rates.

Comparatively, the literature has paid less attention to the impact of inequality on poverty (Bourguignon 2003; Ravallion 2005; Ferreira et al. 2010; Kalwij and Verschoor 2007). This is precisely the mechanism behind the indirect inequality-to-growth channel analyzed in this paper, and not covered in earlier literature. More recently, Sehrawat and Giri (2018), the aforementioned Bergstrom (2020) and Lakner et al. (2020) find evidence supporting the role of declining inequality for poverty reduction.

## Growth, inequality and poverty: data and empirical implementation

We turn to the description of our empirical strategy. First we describe the data and then the econometric approach employed in the estimation.

### Data

Since our focus is not on cyclical growth fluctuations, we follow the empirical literature on inequality and growth and construct a panel data set of non-overlapping 5-year observations on the three variables of interest: inequality, growth and poverty. We focus on the 1960–2010 period, as done by the recent empirical literature on inequality and growth. Growth is measured as the log difference of real per capita income over the entire 5-year interval, while poverty and inequality are measured at the beginning of the interval. This means we only need to collect poverty and inequality data up to 2005.

We use the Gini index to measure inequality and take the UN-WIID2 (2008) database as our primary source of data on income inequality. It includes 5313 surveys for 154 countries from 1950 to 2006. We complete the WIID2 data with information from PovcalNet, which adds another 122 country-year (16 countries) observations over the 1960–2010 period. In a number of instances, there are multiple surveys referring to the same country-year, but they offer different coverage or use different concepts of income. We restrict our sample to Gini indexes based on nationally representative surveys. Moreover, data are sometimes based on income and other times on expenditure figures; income is net of transfers and taxes in some cases and not in others; the unit of analysis may be the individual or the household, etc. To correct at least in part for this heterogeneity, we adjust the original Gini data following Dollar and Kraay (2002).^8^

For economic growth, we use national accounts purchasing-power-parity (PPP)-adjusted per capita GDP data from the Penn World Tables 7.1, the same source used by Berg et al. (2018) and many other studies of inequality and growth, which facilitates comparability with them. Sala-i-Martin (2006) and Dollar and Kraay (2002), among many others, emphasize the advantages of using per capita GDP instead of the mean level of income obtained directly from household surveys. The survey mean usually does not match per capita income from the national accounts, because of differences in concepts and methodology, inconsistent data collection methods, misreporting, etc. Additionally, for many of the country-year observations for which we have information on inequality, we do not have matching information on mean income from the same source, which hampers the construction of a large panel dataset. In contrast, national accounts data are reported yearly for all countries, using a homogenous methodology, which, in addition, allows us to compare our empirical results with those of the ample macroeconomic literature on income inequality and growth.

Regarding poverty data, we follow the strategy proposed by Dollar and Kraay (2002), López and Servén (2015), Sala-i-Martin (2006) and Pinkovskiy and Sala-i-Martin (2013). These authors point out that combining poverty and income growth data from household surveys and national accounts may lead to misleading conclusions, because of the inconsistencies between the two sources just noted. To avoid this problem, they use PWT data to construct both income growth and poverty measures, with the latter computed assuming that household income follows a lognormal distribution. Thus, we construct a set of poverty measures (the headcount ratio P0, the poverty gap P1 and the squared poverty gap P2) using a lognormal approximation on the basis of the observed per capita GDP levels and Gini coefficients.^9^ We also experiment with alternative, widely used poverty lines: US$ 1.25, US$ 2 and US$ 4 per person per day, in 2005 PPP US$ (see Appendix 1 for details).

This approach allows a considerable increase in sample size. Despite the progress made in recent years, mainly through the PovcalNet project, survey-based poverty data are still relatively scarce, at least in comparison with the size of the standard cross-country time-series growth dataset. Using the lognormal approximation, we assemble 746 observations on poverty over non-overlapping 5-year intervals, covering 156 countries between 1960 and 2005 (an average of almost five observations per country).^10^ In contrast, using the January 2020 version of PovcalNet over the same 1960–2005 time span, we can construct a dataset of 383 poverty observations over non-overlapping 5-year intervals for 144 countries, roughly half the size of our sample—i.e., an average of less than 3 observations per country, with data for the vast majority of countries starting in 1990 or later.^11^

As far as we are aware, ours is the largest sample used to date to study the impact of poverty on growth. It exceeds by far the samples used by the two earlier papers analyzing the poverty-growth nexus in a panel regression setting: López and Servén (2015) assemble a sample comprising 325 observations from 85 countries over 1960–2000, while Ravallion (2012) uses unbalanced panel data from PovcalNet covering up to 97 developing countries over a shorter time span, 1981–2005.

Table 1 presents summary statistics on annual growth, mean income, inequality and poverty for the common sample of these variables in the unbalanced 1960–2010 panel. The table shows the wide range of per capita income levels (expressed in 2005 US dollars in PPP terms) in the sample—from just over $200 (the Democratic Republic of Congo in the mid-2000s) to about $73,000 (Luxembourg in 2005). The median observation corresponds to Brazil in the mid-1970s, with per capita income about $5500. The overall sample mean is about $9800, much larger than the median, which reflects a world income distribution skewed to the right.

**Table 1:** Growth, inequality and poverty data: summary statistics

	Median	Mean	Std	P10	P90	Min	Max
GDP per capita growth	0.025	0.025	0.030	− 0.012	0.061	− 0.086	0.201
Real per capita income	5651.1	9792.5	10,462.5	816.4	26,053.7	207.5	73,243.0
Gini coefficient	0.394	0.402	0.100	0.280	0.543	0.157	0.742
P0 (US$ 1.25)	0.005	0.096	0.177	0.000	0.364	0.000	0.906
P0 (US$ 2)	0.023	0.162	0.247	0.000	0.610	0.000	0.969
P0 (US$ 4)	0.130	0.287	0.336	0.000	0.881	0.000	0.999
P1 (US$ 1.25)	0.001	0.040	0.086	0.000	0.146	0.000	0.602
P1 (US$ 2)	0.006	0.073	0.132	0.000	0.270	0.000	0.722
P1 (US$ 4)	0.038	0.151	0.212	0.000	0.523	0.000	0.855
P2 (US$ 1.25)	0.000	0.023	0.056	0.000	0.077	0.000	0.497
P2 (US$ 2)	0.002	0.044	0.089	0.000	0.158	0.000	0.594
P2 (US$ 4)	0.016	0.100	0.156	0.000	0.356	0.000	0.750

Headcount poverty rate (P0); poverty gap (P1); squared poverty gap (P2); alternative poverty lines: US$ 1.25, US$ 2 and US$ 4, per person per day (2005 PPP). Poverty is obtained from a lognormal approximation on the basis of the observed per capita GDP (PWT 7.1) levels and Gini coefficients (UNU-WIDER 2008). See Appendix 1 for details

Regarding inequality, both the median and the mean of the Gini coefficient equal 0.4, which matches the values found for the U.S. (in 2000), Burkina Faso (in 1995), Turkey (in 2010) or Singapore (in 1970). The maximum value (above 0.74) corresponds to Zimbabwe in 1995, and the minimum (below 0.16) corresponds to Bulgaria in 1975. Around 80% of the observations fall in the range between 0.28, a value found among Western European countries, and 0.54, a value found among Latin American and Sub-Saharan African countries.

Poverty rises by construction with the poverty line and declines as the poverty measure changes from P0 to P2 (i.e., as one considers more bottom-sensitive measures). For our lognormal poverty estimates, the table shows that median headcount poverty P0 is 0.6% using US$ 1.25 per day as poverty line, but it raises to 2.3% with a US$ 2 poverty line, and to 13% with US$ 4. Likewise, the median P1 ranges from less than 0.1% for US$ 1.25 to about 4% for US$ 4, while the median P2 ranges from less than 0.1% for US$ 1.25 to almost 2% for US$ 4. Although the mean and the median of these poverty measures are relatively small, the heterogeneity in the sample is quite high, since the ranges of the various poverty measures run from a minimum of zero (reflecting the presence of high-income countries in the sample) to a maximum whose value depends on the particular poverty measure and poverty line under consideration. For example, depending on the poverty lines considered, these maximum levels go from 90 to 99% for P0, from 60 to 86% for P1 and from 50 to 75% for P2. The maximum corresponds in all cases to Tanzania. We use headcount poverty P0 (with a poverty line of US$ 2 per day) as our baseline poverty measure for the rest of the paper.

**Fig. 1:**
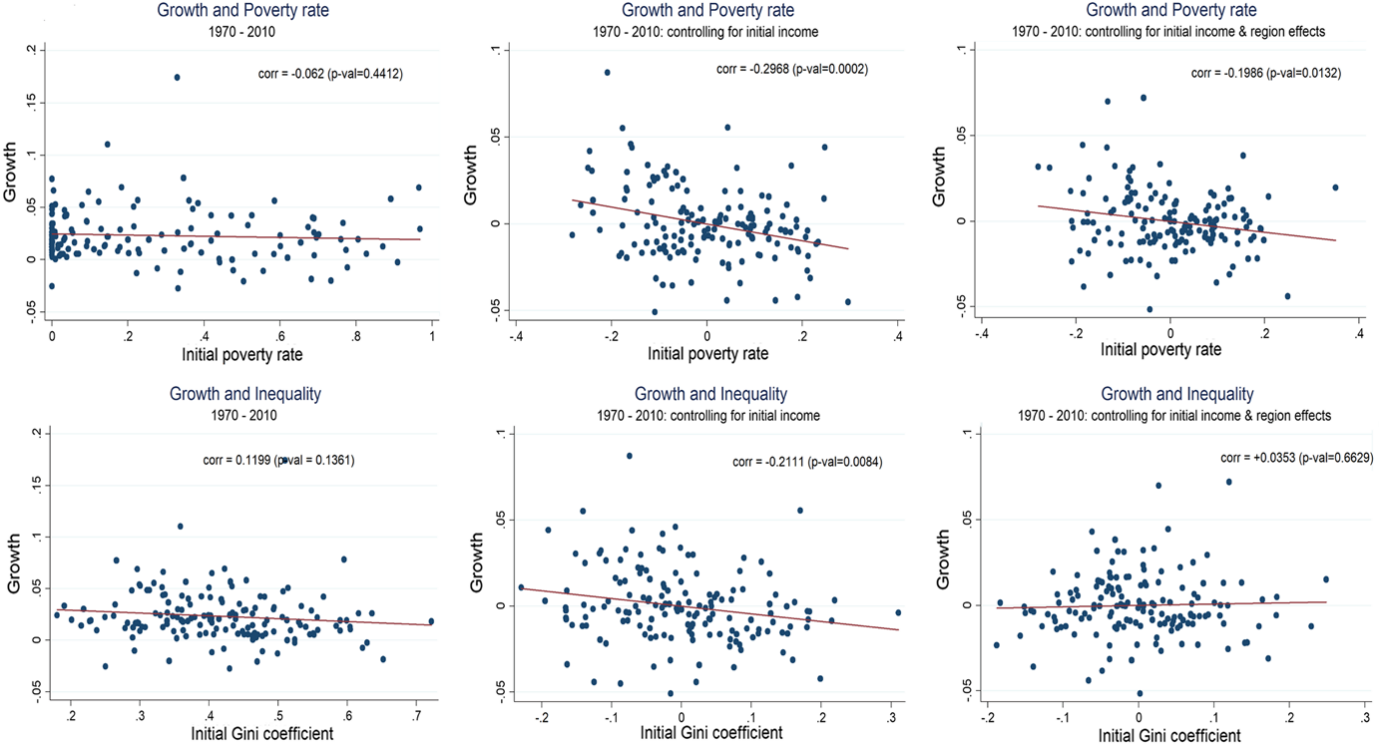
Growth, poverty and inequality: preliminary cross section evidence. *Note* Growth is measured as per capita annual GDP growth between 1970 and 2010. The initial period is 1970. For the graphs in the second column, growth, as well as initial poverty P0, and the initial Gini coefficients G0 are the residuals from projecting the respective original variables on initial per capita GDP (in logs). For the graphs in the third column, they are measured as the residuals from projecting the respective original variables on initial per capita GDP (in logs) and a set of regional dummies (North America, Europe and Central Asia, Latin American and the Caribbean, Middle East and North Africa, Sub-Saharan Africa, South Asia and East Asia and the Pacific)

Figure 1 shows the sample correlation between annual per capita growth, the baseline P0 (for US$ 2) and the Gini coefficient. The top graphs plot growth against lagged poverty, and the bottom graphs plot growth against the Gini coefficient. The leftmost graphs show the unconditional correlation, while the center graphs control for lagged income and the rightmost graphs add also regional dummies. The top left scatter, which shows the unconditional correlation between growth and poverty, highlights the degree of heterogeneity in the sample. For instance, there is a wide range of observations with very small poverty rates and very large variation in growth rates (from − 5% to + 10%). At high poverty rates (above 80%, say), the range of variation in growth rates is fairly wide as well. However, once we control for real per capita GDP (top center graph), the correlation turns negative and significant. The result is robust to the addition of regional dummies. Results are different for the growth-inequality scatter plots. The ambiguous relation shown in the leftmost graph turns negative when we control for real per capita GDP. However, it becomes slightly positive (but insignificant) when adding regional dummies.

Additional controls used in the empirical exercises described in Sects. 4 and 5 (years of schooling, investment prices, inflation, trade openness, government size, degree of democracy, etc.) come from the Penn Word Tables, the World Development Indicators database, the Barro and Lee (2013) educational attainment database, and the political risk module of the International Country Risk Database (ICRD).

The controls used are standard in the empirical growth literature (Perotti 1996; Forbes 2000; Knowles 2005; Barro 2000, among many others). In particular, we consider the price of investment goods relative to that of the USA as a measure of market distortions, so its expected growth impact is negative. As a measure of human capital, we consider the average years of secondary education for males and females in our baseline specification, and the rate of primary and secondary school attainment (as a percentage of the population) in our robustness analysis. The distinction between male and female education is motivated by the finding that the latter appears to be more important than the former in raising labor productivity in developing countries (Owen et al. 2002). Human capital is expected to have a positive impact on growth. However, neither the years of education nor the educational attainment measures capture the quality of education (Hanushek 2017), which detracts from the significance of their growth contribution (Sianesi and Van Reenen 2003). Also, the contribution could be highly nonlinear (Liu and Stengos 1999), so that linear regressions could generate misleading conclusions (a question we revisit in Sect. 5.1).

We also consider standard policy indicators as control variables: the rate of inflation of the GDP deflator as an indicator of macroeconomic stability, the adjusted ratio of the country’s volume of trade to its GDP as an indicator of the degree of openness of the economy,^12^ and the ratio of public consumption to GDP as an indicator of the burden imposed by the government on the economy. As a measure of public infrastructure, we update the composite index constructed by Calderón et al. (2015). It comprises the telecommunication sector (the number of main telephone lines per 1000 workers), the power sector (the electricity generating capacity in MW per 1000 workers), and the transportation sector (the length of the road network—in km. per sq. km. of land area). Finally, we consider controls related to institutional quality, such as the degree of democracy, and government stability.

Table 13 in the Appendix 2 describes all the variables used in the paper (source, sample size, mean and standard deviation), either in the baseline estimation (Sect. 4) or the robustness checks (Sect. 5). In turn, Table 14 reports the pairwise correlation matrix of all controls and core variables in the model (growth, per capita GDP, poverty and inequality). Correlations are shown for the full sample, and they are calculated using the variables transformed as they enter in the regressions (i.e., per capita GDP in logs; poverty and the Gini coefficient in levels; adjusted openness and government size in logs, etc.).

In general, the correlations show the expected signs. In the case of growth, they anticipate the signs of the coefficient estimates obtained below: positive for the education variables, openness, infrastructure, democracy and government stability; negative for investment prices, inflation, and government size, as well as poverty and inequality. In turn, the pairwise correlations between the control variables are generally small except for the human capital variables, ruling out potential collinearity concerns for the regression analysis.

### Empirical strategy

To explore the links between growth, inequality and poverty, we use a specification adding suitable measures of poverty to an otherwise standard empirical growth regression (López and Servén 2015; Ravallion 2012):

1 $$\ln y_{it} - \ln y_{it - 1} = \alpha_{i} + \gamma_{t} + \beta_{0} \ln y_{it - 1} + \delta_{0} p_{it - 1} + \omega^{\prime}x_{it} + \varepsilon_{it} ,$$

where $lny$ is the log of per capita income, $\alpha_{i}$ and $\gamma_{t}$ are country- and time-specific effects, *p* is a measure of poverty, *x* represents a set of control variables, which we shall discuss shortly, and $\varepsilon$ is an i.i.d error term. Likewise, we estimate the standard inequality-growth regression (Forbes 2000; Berg et al. 2018),

2 $$\ln y_{it} - \ln y_{it - 1} = \alpha_{i} + \gamma_{t} + \beta_{1} \ln y_{it - 1} + \varphi_{0} g_{it - 1} + \omega^{\prime}x_{it} + \varepsilon_{it} ,$$

where $g$ is the Gini coefficient. The parameters $\delta_{0}$ and $\varphi_{0}$ in (1) and (2) capture the impacts on growth of poverty and inequality, respectively, given lagged per capita income.

It is important to note that, even if inequality has no direct effect on growth—as assumed in Eq. (1), which omits the Gini coefficient—it can still affect growth indirectly through poverty. The reason is that inequality and poverty are related. This is not due to our assumption of lognormality when constructing the poverty data, but just a general consequence of the very definition of poverty as the share of the population whose income lies below the poverty line. Given the poverty line and the overall population’s mean income, an increase in inequality (more precisely, a mean-preserving spread of the income distribution) must bring more individuals below the poverty line, and therefore raise the poverty rate.^13^ Hence, poverty and inequality are positively correlated in general, a fact that also applies to our dataset, as can be confirmed from the pairwise correlations reported in Table 14. If poverty has a negative effect on growth (i.e., if $\delta_{0}$ in (1) is negative), it follows that an increase in inequality would raise poverty and reduce growth through this indirect channel. We return to this issue in Sect. 6.

Furthermore, if inequality does have a direct effect on growth, the poverty coefficient estimate from a regression equation like (1) that erroneously omits inequality will be biased, and—other things equal—the bias will be greater the larger the correlation between poverty and inequality. To avoid such problem, we also estimate a model including both lagged inequality and lagged poverty as explanatory variables:

3 $$\ln y_{it} - \ln y_{it - 1} = \alpha_{i} + \gamma_{t} + \beta_{2} \ln y_{it - 1} + \delta_{1} p_{it - 1} + \varphi_{1} g_{it - 1} + \omega^{\prime}x_{it} + \varepsilon_{it} .$$

Estimation of (3) merits comment. In principle, $\delta_{1}$ captures the impact on growth of a shock to poverty holding constant inequality and average income, along with the other regressors. Thus, identifiability of $\beta_{2}$, $\delta_{1}$ and $\varphi_{1}$ in a linear regression setting requires that poverty not be an almost exact linear combination of $\ln y$ and $g$—otherwise, the estimating equation would feature (nearly) perfect collinearity. In our data set, panel regressions of poverty on per capita income and the Gini coefficient account for less than half of the sample variation in poverty.^14^ Thus, collinearity does not prevent identification of $\beta_{2}$, $\delta_{1}$ and $\varphi_{1}$ in (3), as more than half of the sample variation in poverty can be attributed to shocks uncorrelated with average income or inequality.^15^

We turn to the set of controls included in *x*. Rather than adding to the already huge variety of empirical growth models contributing yet another idiosyncratic set of regressors, we opt for considering alternative growth specifications found in the literature, in order to explore the sensitivity of our results to the specific choice of control variables. We use the four models described next as our baseline specifications, leaving for Sect. 5 a robustness check on the inclusion of additional controls.

First, we consider a skeleton model of growth (M1), which includes only lagged income, poverty and the Gini coefficient as regressors. In this setting, the estimated parameters capture the direct impacts of poverty and inequality on growth, as well as potential indirect effects due to other variables omitted from the model (Galor 2009). Our second model (M2) is taken from the empirical literature on inequality and growth (Perotti 1996; Forbes 2000). It comprises a measure of market distortions (the domestic price of investment goods relative to that of the USA) and a measure of human capital, given by the average years of secondary education of the male and female populations, considered separately. Our third model (M3) focuses on standard policy indicators (Barro 2000). It includes the rate of inflation of the GDP deflator (macroeconomic stability), the adjusted ratio of the country’s volume of trade to its GDP (the degree of openness), and the ratio of public consumption to GDP (government size). Lastly, the fourth model (M4) is taken from López and Servén (2015). It includes the inflation rate, the average years of secondary female education, and a lagged composite index of public infrastructure.

Our empirical strategy has to confront two endogeneity concerns. On the one hand, the joint determination of income, poverty and inequality could result in biased estimates. The fact that poverty and inequality are pre-determined in (1)–(3) should help alleviate, even if not necessarily eliminate, this concern. On the other hand, the country-specific unobservable *α*_*i*_ may be correlated with the regressors in (1)–(3).

Dealing with endogeneity requires an instrumental variable estimation approach. However, we have no obvious candidates for suitable external instruments—i.e., exogenous variables correlated with poverty and/or inequality but not with growth. Thus, following common practice in the empirical literature on the effects of inequality on growth, we opt for using GMM panel estimators employing “internal instruments,” that is, instruments based on lagged values of the explanatory variables. To build such instruments, we assume that the explanatory variables (including poverty and inequality) are weakly exogenous. In other words, they can be affected by current and past realizations of the growth rate—e.g., today’s poverty or inequality may depend on past growth—but must be uncorrelated with future realizations of the time-varying growth shock. This assumption does not seem particularly restrictive; furthermore, we can statistically examine its validity through several specification tests, as explained below.

Specifically, we take first differences in (1)–(3) to remove the country-specific unobservable *α*_*i*_. This leaves us with the first-differenced time-varying residual (e.g., $\varepsilon_{it} - \varepsilon_{it - 1}$) in the transformed equations. Under the assumption that the original regressors are weakly exogenous, so that for any regressor $z$ we have $E\left[ {z_{is} \varepsilon_{it} } \right] = 0$ for $s < t$, the levels of the regressors lagged two or more periods become valid instruments for GMM estimation of the parameters of the first-differenced equations, because $E\left[ {z_{it - s} \left( {\varepsilon_{it} - \varepsilon_{it - 1} } \right)} \right] = 0$ for $s > 1$. Using these instruments, we can consistently estimate the parameters of interest, namely *β, δ,* and *φ*, even though the dependent variable of the first-differenced equations is the change in the growth rate, rather than the growth rate itself.^16^

However, working only with the model in first differences may lead to major finite sample biases if the variables are highly persistent, because their lagged levels become weak instruments for the first-differenced regressors (Blundell and Bond 1998). Under the additional stationarity assumption that $E\left[ {\left( {z_{it} - z_{is} } \right)\alpha_{i} } \right] = 0$ for all $t$ and $s$, *differences* of the regressors lagged one or more periods become valid instruments for the original level Eqs. (1)–(3) (Blundell and Bond 1998). This allows building the so-called system GMM estimator, which estimates the parameters of interest combining the first-differenced equation and the original levels equation.

The GMM estimator is consistent as long as the underlying instruments are valid. Their validity can be tested using Hansen’s *J* test of over-identification. We also report results for the Difference-in-Hansen statistic, which tests the validity of the subset of instruments employed in the level equation of the system GMM estimation.

A problem often encountered in GMM estimation is the excessive proliferation of instruments, which biases downward the estimated standard errors and weakens the power of the over-identification tests (Roodman 2009). To remedy this, we apply the Windmeijer (2005) correction to the variance–covariance matrix and also reduce the number of instruments employed in the estimation (Roodman 2009). Specifically, we limit the number of lags in the matrix of instruments, and/or collapse the matrix of instruments and create one instrument for each variable and lag distance, rather than one instrument for each lag distance, time period and variable as commonly done in the system GMM approach.

Although the GMM estimators attempt to deal with the endogeneity of regressors typical of dynamic panel data models like (1)–(3), when the cross-sectional dimension of the sample is not large relative to its time dimension—a common situation with macroeconomic panel data—these GMM estimators can behave poorly (Bun and Sarafidis 2015). In this setting, it is not obvious that GMM should be preferred to more conventional estimation methods, such as OLS with time and/or country dummies. Our sample should not be affected by this problem, since its cross-sectional dimension is much larger than its time dimension. Nevertheless, in the next section, we report both sets of estimates, which helps also assess the robustness of the results.

## Empirical results: baseline model and specification

We next report the main empirical results and assess the robustness of the poverty-growth and inequality-growth relationships. Tables 2 and 3 present pooled-OLS and within-group (WG) estimates, respectively. We use P0 (with a poverty line of US$ 2) as our baseline measure of poverty.^17^

**Table 2:** Growth, poverty and inequality: panel OLS estimates

	M1. Skeleton model			M2. Extended with education and inv. prices			M3. Extended with policy variables			M4. Extended with policy and infrastructures		
P0, lag	− 0.0450*** (− 5.70)		− 0.0440*** (− 5.71)	− 0.0328*** (− 3.88)		− 0.0334*** (− 4.02)	− 0.0382*** (− 4.58)		− 0.0380*** (− 4.70)	− 0.0424*** (− 3.84)		− 0.0430*** (− 4.03)
Gini, lag		− 0.0415*** (− 3.63)	− 0.0393*** (− 3.50)		− 0.0252** (− 2.12)	− 0.0266** (− 2.31)		− 0.0399*** (− 3.35)	− 0.0396*** (− 3.36)		− 0.0354** (− 2.44)	− 0.0366*** (− 2.60)
log *y*, lag	− 0.00781*** (− 5.43)	− 0.00143 (− 1.61)	− 0.00873*** (− 5.88)	− 0.00892*** (− 4.80)	− 0.00381*** (− 3.08)	− 0.00952*** (− 5.05)	− 0.00803*** (− 5.43)	− 0.00303*** (− 3.20)	− 0.00920*** (− 5.97)	− 0.0213*** (− 6.13)	− 0.0143*** (− 4.18)	− 0.0215*** (− 6.31)
Inv. deflator, lag				− 0.00482** (− 2.23)	− 0.00629** (− 2.20)	− 0.00453* (− 1.91)						
Female educ., lag				− 0.00299 (− 1.16)	− 0.00300 (− 1.09)	− 0.00176 (− 0.66)				0.00560*** (3.79)	0.00370** (2.44)	0.00509*** (3.48)
Male educ., lag				0.00747*** (2.89)	0.00671** (2.40)	0.00567** (2.13)						
Inflation							− 0.00728 (− 1.41)	− 0.00409 (− 0.84)	− 0.00728 (− 1.41)	− 0.0232*** (− 3.66)	− 0.0165** (− 2.58)	− 0.0217*** (− 3.31)
Trade openness (log)							0.0113*** (4.25)	0.0136*** (5.05)	0.0115*** (4.34)			
Gov. size (log)							− 0.00110 (− 0.40)	− 0.00191 (− 0.67)	− 0.00135 (− 0.48)			
Infrastructure, lag										0.00847*** (2.94)	0.00830*** (2.78)	0.00754*** (2.70)
Num. obs	745	745	745	676	676	676	656	656	656	477	477	477
R2-adjusted	0.096	0.072	0.112	0.124	0.108	0.130	0.120	0.107	0.135	0.149	0.125	0.161

Unbalanced panel with data at 5-year intervals over 1960–2010. The dependent variable is the annual growth rate of per capita GDP. The explanatory variables are real per capita GDP (in logs), the headcount poverty rate (P0) using US$ 2 as poverty line, the Gini coefficient, and alternative sets of additional controls that vary across models M1 (skeleton model), M2 (education and investment prices), M3 (policy variables) and M4 (policy variables and infrastructures). Explanatory variables are all lagged one period (5 years), with the exception of the policy variables in models M3 and M4, which are taken as contemporaneous 5-year averages. A constant term and time dummies are included in all models. Robust *t* statistics in parentheses: ***denotes significance at 1%, **at 5%, *at 10%

**Table 3:** Growth, poverty and inequality: within-group estimates

	M1. Skeleton model			M2. Extended with education and inv. prices			M3. Extended with policy variables			M4. Extended with policy and infrastructures		
P0, lag	− 0.0665*** (− 3.79)		− 0.0764*** (− 4.45)	− 0.0664*** (− 3.89)		− 0.0792*** (− 4.65)	− 0.0716*** (− 3.86)		− 0.0861*** (− 4.53)	− 0.0514** (− 2.46)		− 0.0633*** (− 2.82)
Gini, lag		0.0378 (1.27)	0.0643** (2.27)		0.0454 (1.46)	0.0742** (2.46)		0.0576** (2.00)	0.0865*** (2.98)		0.0457 (1.48)	0.0641** (2.05)
log *y*, lag	− 0.0409*** (− 6.28)	− 0.0303*** (− 4.91)	− 0.0429*** (− 6.18)	− 0.0394*** (− 5.51)	− 0.0256*** (− 4.02)	− 0.0426*** (− 5.86)	− 0.0667*** (− 8.31)	− 0.0557*** (− 6.62)	− 0.0709*** (− 8.38)	− 0.0643*** (− 8.11)	− 0.0580*** (− 8.04)	− 0.0674*** (− 8.18)
Inv. deflator, lag				− 0.00916** (− 2.33)	− 0.0116** (− 2.33)	− 0.00899** (− 2.31)						
Female educ., lag				− 0.00122 (− 0.15)	− 0.0124 (− 1.63)	− 0.00196 (− 0.26)				0.00471* (1.79)	0.00261 (1.04)	0.00603** (2.39)
Male educ., lag				0.00637 (0.75)	0.0148* (1.85)	0.00935 (1.15)						
Inflation							− 0.0224*** (− 3.87)	− 0.0221*** (− 3.87)	− 0.0219*** (− 3.72)	− 0.0368*** (− 4.93)	− 0.0369*** (− 5.45)	− 0.0366*** (− 5.10)
Trade openness (log)							0.0258*** (3.50)	0.0312*** (3.57)	0.0238*** (3.49)			
Gov. size (log)							− 0.0237*** (− 2.94)	− 0.0196** (− 2.57)	− 0.0251*** (− 3.26)			
Infrastructure, lag										0.0177*** (3.75)	0.0236*** (5.90)	0.0170*** (3.44)
Num. obs	745	745	745	676	676	676	656	656	656	477	477	477
R2-adjusted	0.202	0.171	0.216	0.218	0.192	0.236	0.325	0.302	0.348	0.336	0.329	0.350
Num. countries	156	156	156	131	131	131	147	147	147	88	88	88

See note in Table 2

It can be seen that the estimated coefficients of poverty do not change significantly when also including inequality in the model. A quick look at Tables 2 and 3 shows that the coefficient on poverty is negative and significant in all cases. Its magnitude is larger in absolute value in the WG regressions than in the pooled-OLS regressions, but it is in all cases economically significant. Other things equal, a one-standard deviation decline in poverty (24.7 p.p., Table 1) is associated with an increase in income growth between 0.8% and 2.1% per annum. In contrast, results are not robust regarding the inequality-growth relationship. The estimated coefficients on the Gini index are uniformly negative and significant when using pooled-OLS, but uniformly positive in the WG estimation, and significantly so when poverty is also included in the regression (except for model M3, where the Gini index is also significant when poverty is omitted).

The coefficients of the other controls are generally consistent across estimation methods. Lagged income carries negative and significant coefficients in most cases. The market distortions proxy (in model M2) and inflation (M2 and M3) both carry significant negative coefficients. Trade openness (in M3) and the infrastructure index (in M4) carry positive and significant coefficients (Calderón et al. 2015). In contrast, the effects of male and female secondary education depend on model specification. Female education carries a positive and significant coefficient in M4, but turns insignificant in M2, while the coefficient of male education is generally positive. Similarly, among the policy variables, the coefficient of government size is generally negative, but it is significant only for the WG estimates.

Table 4 shows estimation results for first-difference GMM, while Table 5 shows the results for the baseline system GMM specification (limiting the instrument matrix to two lags). In Appendix 3 (Tables 15 and 16), we report results under alternative approaches to reducing the dimension of the system GMM instrument set: collapsing the matrix of instruments while using all lags as instruments (Table 15), and limiting them to two lags and collapsing the instruments at the same time (Table 16). For first-difference GMM (Table 4), we use three lags in the matrix of instruments so as to have the same number of orthogonality conditions as in the baseline system GMM estimation, thus making the results more easily comparable.^18^ The *p* values of the Hansen tests suggest that in virtually every case, the null of joint validity of all instruments cannot be rejected. Moreover, the Difference-in-Hansen test results, whose *p* values always exceed 0.10, point toward the superiority of system GMM over first-difference GMM.

**Table 4:** Growth, poverty and inequality: first-difference GMM estimates

	M1. Skeleton model			M2. Extended with education and inv. prices			M3. Extended with policy variables			M4. Extended with policy and infrastructures		
P0, lag	− 0.0941*** (− 2.59)		− 0.0981*** (− 2.63)	− 0.150*** (− 3.35)		− 0.150*** (− 5.16)	− 0.0997* (− 1.79)		− 0.0947** (− 2.19)	− 0.117** (− 2.25)		− 0.103*** (− 2.75)
Gini, lag		0.0253 (0.37)	0.113 (1.10)		0.0330 (0.47)	0.131** (2.01)		0.0690 (0.73)	0.112 (1.56)		0.0434 (0.43)	0.115 (1.32)
log *y*, lag	− 0.106*** (− 4.15)	− 0.0876*** (− 3.89)	− 0.119*** (− 5.45)	− 0.100*** (− 3.48)	− 0.0756*** (− 4.60)	− 0.0948*** (− 4.47)	− 0.154*** (− 3.71)	− 0.105*** (− 4.50)	− 0.149*** (− 4.47)	− 0.103*** (− 3.24)	− 0.0883*** (− 4.01)	− 0.106*** (− 5.25)
Inv. deflator, lag				− 0.0117 (− 1.19)	− 0.0177 (− 1.47)	− 0.00942 (− 0.90)						
Female educ., lag				0.0616** (2.39)	0.0148 (0.82)	0.0465*** (2.70)				0.00688 (0.71)	− 0.00271 (− 0.20)	0.00611 (0.57)
Male educ., lag				− 0.0525* (− 1.92)	− 0.0140 (− 0.59)	− 0.0319 (− 1.51)						
Inflation							0.0005** (2.51)	0.0005** (2.26)	0.0004* (1.91)	0.0001 (0.06)	0.0017 (0.98)	0.0009 (0.83)
Trade openness (log)							0.0396 (1.56)	0.0532** (2.24)	0.0457* (1.95)			
Gov. size (log)							− 0.0265 (− 1.50)	− 0.0350 (− 1.51)	− 0.0407** (− 2.05)			
Infrastructure, lag										0.00750 (0.37)	0.0245* (1.78)	0.00778 (0.42)
m2-test (*p* value)	0.854	0.924	0.568	0.565	0.503	0.336	0.505	0.833	0.750	0.658	0.641	0.622
AR(3) (*p* value)	0.0261	0.00635	0.0238	0.117	0.102	0.416	0.186	0.155	0.264	0.142	0.146	0.187
Hansen (*p* value)	0.167	0.199	0.0571	0.412	0.269	0.330	0.581	0.431	0.759	0.397	0.575	0.424
Num. obs	502	503	502	467	468	467	248	249	248	345	346	345
Num. countries	130	130	130	113	113	113	84	84	84	79	79	79
Num. instruments	39	39	54	84	84	99	59	59	70	47	47	55

See Note in Table 2. Estimations are done using 2-step first-difference GMM reducing the number of instrument lags to three. The instrument set starts at *t* − 3, and the variance covariance matrix is computed using the small sample correction of Windmeijer (2005). Robust *t* statistics in parentheses. ***denotes significance at 1%, **at 5%, *at 10%

**Table 5:** Growth, poverty and inequality: system GMM estimates

	M1. Skeleton model			M2. Extended with education and inv. prices			M3. Extended with policy variables			M4. Extended with policy and infrastructures		
P0, lag	− 0.117*** (− 3.81)		− 0.121*** (− 5.34)	− 0.0883*** (− 3.71)		− 0.0846*** (− 4.12)	− 0.0666*** (− 2.96)		− 0.0697*** (− 3.85)	− 0.0506* (− 1.88)		− 0.0503** (− 2.32)
Gini, lag		− 0.107*** (− 2.92)	− 0.0955** (− 2.12)		− 0.0553 (− 1.44)	− 0.0587 (− 1.57)		− 0.0908*** (− 3.30)	− 0.106*** (− 4.10)		− 0.00339 (− 0.08)	− 0.0346 (− 1.25)
log *y*, lag	− 0.0200*** (− 4.60)	− 0.00156 (− 0.61)	− 0.0228*** (− 6.19)	− 0.0166*** (− 4.61)	− 0.00210 (− 0.92)	− 0.0173*** (− 5.30)	− 0.0160*** (− 4.72)	− 0.00653** (− 2.50)	− 0.0184*** (− 5.95)	− 0.0362*** (− 4.72)	− 0.0168 (− 1.53)	− 0.0299*** (− 3.29)
Inv. deflator, lag				− 0.00137 (− 1.38)	− 0.00339* (− 1.93)	− 0.000830 (− 0.70)						
Female educ., lag				0.00122 (0.20)	0.00267 (0.44)	0.00385 (0.57)				0.00714** (2.47)	0.000557 (0.15)	0.00557* (1.83)
Male educ., lag				0.00241 (0.36)	− 0.00158 (− 0.23)	− 0.00144 (− 0.19)						
Inflation							0.001* (1.93)	0.0012*** (2.91)	0.001*** (2.60)	0.0004 (0.68)	0.001** (2.02)	0.001* (1.83)
Trade openness (log)							0.0255*** (3.26)	0.0264*** (2.83)	0.0217*** (2.58)			
Gov. size (log)							− 0.00306 (− 0.42)	− 0.00861 (− 0.91)	− 0.00349 (− 0.44)			
Infrastructure, lag										0.0222*** (3.07)	0.0189** (2.03)	0.0166** (2.01)
m2-test (*p* value)	0.108	0.223	0.215	0.0574	0.117	0.117	0.279	0.434	0.493	0.227	0.203	0.313
AR(3) (*p* value)	0.942	0.659	0.671	0.735	0.729	0.785	0.622	0.609	0.474	0.845	0.773	0.746
Hansen (*p* value)	0.138	0.160	0.279	0.225	0.242	0.572	0.224	0.295	0.616	0.163	0.269	0.477
Diff-Hansen for levels (*p* value)	0.220	0.640	0.248	0.382	0.490	0.848	0.375	0.662	0.783	0.372	0.617	0.850
Num. obs	745	745	745	676	676	676	656	656	656	477	477	477
Num. countries	156	156	156	131	131	131	147	147	147	88	88	88
Num. instruments	54	54	76	120	120	142	116	116	138	82	82	97

See note Table 4 Estimations are done using 2-step system GMM reducing the number of instrument lags to two. The instrument set starts at *t* − 3, and the variance covariance matrix is computed using the small sample correction of Windmeijer (2005). The difference Hansen test assesses the validity of the instruments for the level equation in system GMM. Robust *t* statistics in parentheses. *** denotes significance at 1%, ** at 5%, * at 10%

The parameter estimates of the variables of interest follow the same pattern found earlier. The coefficient on the poverty headcount is consistently negative and highly significant, regardless of the choice of model and specification. In contrast, the coefficient of the inequality variable varies in sign and significance depending on the GMM approach and the controls used in the estimation. It is always positive and in one case significant for first-difference GMM, consistent with our results for the WG estimates in Table 3 and part of the earlier literature (e.g., Forbes 2000). However, it is negative and, in some cases, significant for system GMM, consistent with our results for pooled-OLS and another strand of the literature (e.g., Berg et al. 2018, and references therein). The negative effect of poverty on growth is robust to changes in model specification and estimation method, while the effect of inequality on growth, which has been the focus of a massive literature, is not.

The theoretical model outlined in López and Servén (2015) and explored in Marrero and Servén (2018) helps rationalize our empirical results. In that model, poor individuals—i.e., those whose initial endowment is below a minimum consumption level—do not save and do not contribute to the economy’s aggregate growth. In the absence of financial markets, the model shows that poverty is unambiguously growth-deterring, while inequality can affect growth directly, through the savings of the non-poor, and indirectly, through its effect on poverty. While the indirect effect is negative, the direct effect is ambiguous (as found by the empirical literature), and so is the overall impact of inequality on growth.

As a further diagnostic check on the GMM estimates of Tables 4, 5, 15, 16, we inspected the residuals for cross-sectional dependence, using Pesaran’s (2021) CD test, and focusing on the model versions including both poverty and inequality. Results are shown in Table 17 (Appendix 4). In the majority of cases, the test results are supportive of the empirical specification. This is particularly the case for the models including policy variables (models M3 and M4 in the aforementioned tables), for which the test fails in all cases to reject the null of cross-sectional independence. For the stripped-down model M1, which omits all controls, results are more mixed, as the test fails to reject the null at the conventional 5% level in some exercises (those in Tables 4 and 5) but rejects it in others (those in Tables 15, 16). The exception is model M2, for which the test consistently finds significant evidence of cross-sectional dependence.^19^ Overall, we take these results as supporting the view that models M3 and M4 are correctly specified. However, the presence of residual cross-sectional correlation in model M2—first explored by Perotti (1996) and Forbes (2000), suggests that the model’s estimated standard errors may be incorrect.^20^

### Weak instruments analysis

Bazzi and Clemens (2013) have raised the potential problem of weak instruments when using system GMM estimation in growth regressions. Weak identification arises when the instruments are only weakly correlated with the endogenous regressors, and its consequence is that estimators perform poorly (Nelson and Startz 1990). To assess the strength of the instruments employed in our system GMM estimations—in particular, the identification of the poverty and inequality parameters—we use tools designed for settings featuring multiple endogenous regressors. We follow Sanderson and Windmeijer (2016) (SW hereafter), who propose a conditional *F* statistic based on Angrist and Pischke (2009) to test whether, in a multivariate setting, a particular endogenous regressor is weakly instrumented. For each such regressor, a conditional test is constructed by “partialing-out” linear projections of the remaining endogenous regressors. SW show that the conditional *F* statistic can be assessed against the Stock and Yogo critical values, and the weakness can then be expressed in terms of the size of the bias of the IV (or 2SLS) estimator relative to that of the OLS estimator. The null hypothesis is that the instruments are weak. It is rejected if the conditional *F* statistic exceeds the corresponding critical value, and we use a critical value allowing for a 30 percent maximal relative bias. We also perform a Chi-square under-identification test separately for each regressor. Here, the null hypothesis is that the matrix of coefficients from the first‐stage conditional regressions is not full rank, signaling a complete failure of identification. Thus, rejection of the null supports identification, although not necessarily the absence of weak identification (Kleibergen and Paap 2006).

These tests have been originally designed for use with external instruments in IV or 2SLS settings; no suitable equivalents exist for system GMM at present. Thus, to apply the tests to our system GMM setting, we follow Bun and Windmeijer (2010) and construct the exact instrument matrix for the difference and level equations of each system GMM estimator, and then apply the standard 2SLS regressions and tests to each case.Table 6 reports the results of the SW tests for all models estimated under our baseline system GMM specification (Table 5). For lagged poverty and inequality, we present the Chi-square under-identification test, and the weak instruments *F* statistic.

**Table 6:** Weak instruments analysis for the baseline system GMM estimations

	M1. Skeleton model			M2. Extended with education and inv. prices			M3. Extended with policy variables			M4. Extended with policy and infrastructures		
	Only P0	Only Gini	P0 and Gini	Only P0	Only Gini	P0 and Gini	Only P0	Only Gini	P0 and Gini	Only P0	Only Gini	P0 and Gini
A. First-difference equation												
A.1. Under-identification test												
SW Chi-2 (*p* val) (P0, lag)	0.00	–	0.00	0.00	–	0.00	0.00	–	0.00	0.00	–	0.00
SW Chi-2 (*p* val) (Gini, lag)	–	0.00	0.05	–	0.00	0.00	–	0.00	0.00	–	0.00	0.00
A.2. Weak identification Test												
SW *F*-stat (P0, lag)	8.27	–	6.16	5.41	–	4.34	33.62	–	20.14	5.89	–	7.23
SW *F*-stat (Gini, lag)	–	1.69	1.23	–	3.04	2.36	–	19.98	10.82	–	3.19	3.16
Stock-Yogo, 30% maximal IV relative bias	4.29	4.29	4.13	3.96	3.96	3.91	3.97	3.97	3.91	4.18	4.18	4.11
B. Level equation												
B.1. Under-identification test												
SW Chi-2 (*p* val) (P0, lag)	0.00	–	0.00	0.00	–	0.00	0.00	–	0.00	0.00	–	0.00
SW Chi-2 (*p* val) (Gini, lag)	–	0.00	0.00	–	0.00	0.00	–	0.00	0.00	–	0.00	0.00
B.2. Weak identification Test												
SW *F*-stat (P0, lag)	6.57	–	5.34	5.14	–	4.62	13.69	–	11.88	14.32	–	10.56
SW *F*-stat (Gini, lag)	–	7.57	5.60	–	4.87	4.38	–	15.28	13.51	–	9.45	7.69
Stock-Yogo, 30% maximal IV relative bias	4.67	4.67	4.46	4.23	4.23	4.15	4.24	4.24	4.16	4.24	4.24	4.16

This table reports weak instruments tests for results in Table 6: a weak instruments *F* test and a Chi-2 under-identification tests allowing for separate instruments diagnosis (Angrist and Pischke 2009). See Bun and Windmeijer (2010). An *F* statistic below the reference value in Stock and Yogo (2005) represents evidence that the coefficient estimate of the variable under consideration suffers from a weak instruments problem

The Chi-square tests indicate that under-identification of the coefficients on poverty and the Gini index is not a major problem in any of the models and specifications considered, neither for the level equation nor for the difference equation. As for the SW weak instruments *F* test, the null hypothesis that lagged poverty and weakly instrumented is rejected, as the conditional *F* statistic exceeds the Stock and Yogo critical value for both the first-difference and the level equations in all cases. In contrast, the null that lagged inequality and weakly instrumented is not rejected for the first-difference equation in models M1, M2 and M4, while it is rejected in all other situations.

Overall, the results of these tests suggest that instrument weakness is not a major problem with our estimates. Further, we also conclude that including the level equation in the GMM estimation helps alleviate potential problems of weak instruments, especially when estimating the effect of inequality on growth. This points to system GMM as the preferred estimation approach.

## Estimation results: robustness analysis

We next perform an extensive set of robustness checks, along five dimensions. First, we allow for nonlinearities using a nonparametric approach. Second, we consider alternative poverty measures. Third, we replace our poverty data with the PovcalNet data. Fourth, we assess additional control variables. And fifth, we consider alternative econometric specifications.

### Nonlinearities: nonparametric analysis

One potential concern with our linear regression analysis is that the estimated effect of poverty on growth could be partly capturing nonlinearities in the relationship between growth and other controls. Following Liu and Stengos (1999), we use the Baltagi and Li’s (2002) semiparametric fixed-effects regression estimator to assess this question. This approach considers a linear fixed-effects model such as our Eqs. (1)–(3) allowing for a nonparametric specification for one particular regressor.^21^

**Fig. 2:**
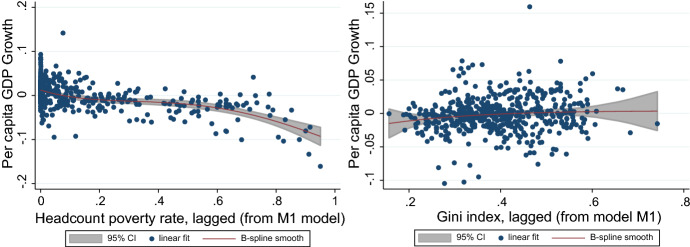
Poverty, inequality and growth: nonlinear nonparametric estimates. *Note* Estimations made using the Baltagi and Li’s (2002) semiparametric fixed-effects regression estimator. We estimate Eq. (3) for model M1 allowing for a nonparametric (approximated by a spline interpolation) specification for one particular regressor at a time. In this case: lagged poverty (left graphic) and lagged Gini index (right graphic)

Figure 2 depicts the nonlinear nonparametric estimates of the effects of lagged poverty and the lagged Gini index. To save space, we only show results for model M1, but results using models M2, M3 and M4 are qualitatively similar. They are consistent with our findings using the conventional specification: First, lagged poverty is negatively correlated with growth, and more strongly so for high poverty levels; second, the lagged Gini index is weakly correlated with growth, which echoes the lack of robustness found with the conventional specification. When the Gini index is nonparametrically adjusted, the estimated linear coefficient for poverty is still negative and significant at 5%.

Next, we use the same procedure to assess nonlinearities for all the other regressors in models M2, M3 and M4, taken one at a time. Table 18 in Appendix 5 reports the resulting coefficient estimates on inequality, poverty, and lagged income obtained in this manner. These estimates can be compared with the WG estimates in Table 3 and the first-difference GMM estimates in Table 4.

Our conclusion that poverty is growth-deterring does not change when using nonlinear nonparametric specifications for any regressor. Moreover, like in our WG and first-difference GMM specifications, the parameter estimates on the Gini coefficient are positive and significant in the majority of the cases. In Fig. 4 in Appendix 5, we graph the estimates of the nonlinear components for average years of male and female education, the two variables for which we find a significant nonlinear relationship, as in Liu and Stengos (1999). For female education, there is a clear positive nonlinear relationship: after 2 years of average female education, the effect on per capita GDP growth turns positive and keeps rising until the 4–5 years mark; for average years of male education, the nonlinear relationship is more concave than for female education, and the slope is positive for almost all years, with the exception of some observations above the 6-year mark. For comparison, we report also the results obtained with government size, which yields a close to linear negative slope, and the infrastructure index, which yields a close to linear positive slope.

### Alternative poverty measures and poverty lines

To assess the robustness of our results to the use of alternative poverty measures and poverty lines, we re-estimate the empirical growth equations using the poverty gap (P1) and the squared poverty gap (P2), and considering alternative poverty lines: US$ 1.25, $2 and $4 per person per day. The poverty rates based on alternative poverty lines and poverty measures exhibit high, but not perfect, pairwise correlation (ranging from 0.74 to 0.99). As the different poverty measures capture different dimensions of poverty, the robustness analysis can be informative about potential differences in their respective effects on growth.

Table 7 reports system GMM estimates of all specifications that this strategy yields, with the matrix of instruments defined as in Table 5. Regarding the poverty coefficient, 72 out of 72 estimates are negative, and 71 out of 72 are also significant, regardless of the choice of poverty measure, poverty line, and set of control variables employed. In general, the absolute value of the poverty coefficient rises as we move from P0 to P2. In turn, while all 72 estimates of the inequality coefficient are negative (recall that the positive estimates arise from the within-country dimension of the data, Tables 3, 4), only 36 of them are significant at the 10 percent level or better. Finally, the Hansen tests do not show evidence against the validity of the instruments, and the *p* values of the Hansen-difference test (omitted from the table to save space) exceed 0.1 in all cases.

**Table 7:** Estimation results: alternative poverty lines and poverty measures baseline system GMM

	M1. Skeleton model			M2. Extended with education and inv. prices			M3. Extended with policy variables			M4. Extended with policy and infrastructures		
*Headcount poverty rate, P0, poverty line = US$ 1.25*												
P0, lag	− 0.124*** (− 3.49)		− 0.115*** (− 4.67)	− 0.0822*** (− 3.62)		− 0.0804*** (− 3.73)	− 0.0938*** (− 3.35)		− 0.0796*** (− 3.76)	− 0.0606** (− 1.96)		− 0.0584*** (− 2.71)
Gini, lag		− 0.107*** (− 2.92)	− 0.0882** (− 2.18)		− 0.0553 (− 1.44)	− 0.0594* (− 1.69)		− 0.0908*** (− 3.30)	− 0.0960*** (− 3.74)		− 0.00339 (− 0.08)	− 0.0346 (− 1.46)
Hansen (*p* value)	0.212	0.160	0.284	0.235	0.242	0.548	0.172	0.295	0.482	0.134	0.269	0.499
*Headcount Poverty Rate, P0, Poverty line = US$ 2.0*												
P0, lag	− 0.117*** (− 3.81)		− 0.121*** (− 5.34)	− 0.0883*** (− 3.71)		− 0.0846*** (− 4.12)	− 0.0666*** (− 2.96)		− 0.0697*** (− 3.85)	− 0.0506* (− 1.88)		− 0.0503** (− 2.32)
Gini, lag		− 0.107*** (− 2.92)	− 0.0955** (− 2.12)		− 0.0553 (− 1.44)	− 0.0587 (− 1.57)		− 0.0908*** (− 3.30)	− 0.106*** (− 4.10)		− 0.00339 (− 0.08)	− 0.0346 (− 1.25)
Hansen (*p* value)	0.138	0.160	0.279	0.225	0.242	0.572	0.224	0.295	0.616	0.163	0.269	0.477
*Headcount Poverty Rate, P0, Poverty line = US$ 4.0*												
P0, lag	− 0.142*** (− 4.86)		− 0.152*** (− 6.15)	− 0.101*** (− 4.93)		− 0.0944*** (− 4.78)	− 0.0652*** (− 3.12)		− 0.0765*** (− 4.41)	− 0.0557 (− 1.54)		− 0.0629*** (− 2.62)
Gini, lag		− 0.107*** (− 2.92)	− 0.0689 (− 1.55)		− 0.0553 (− 1.44)	− 0.0412 (− 1.24)		− 0.0908*** (− 3.30)	− 0.107*** (− 3.74)		− 0.00339 (− 0.08)	− 0.0206 (− 0.58)
Hansen (*p* value)	0.124	0.160	0.207	0.292	0.242	0.688	0.195	0.295	0.618	0.181	0.269	0.543
*Poverty Gap, P1, Poverty line = US$ 1.25*												
P1, lag	− 0.263*** (− 3.74)		− 0.227*** (− 4.57)	− 0.145*** (− 3.35)		− 0.138*** (− 2.90)	− 0.200*** (− 4.89)		− 0.158*** (− 4.49)	− 0.144** (− 2.28)		− 0.121** (− 2.45)
Gini, lag		− 0.107*** (− 2.92)	− 0.0658* (− 1.66)		− 0.0553 (− 1.44)	− 0.0486 (− 1.21)		− 0.0908*** (− 3.30)	− 0.0789*** (− 2.63)		− 0.00339 (− 0.08)	− 0.0182 (− 0.57)
Hansen (*p* value)	0.319	0.160	0.346	0.184	0.242	0.620	0.194	0.295	0.520	0.129	0.269	0.630
*Poverty gap, P1, poverty line = US$ 2.0*												
P1, lag	− 0.191*** (− 3.65)		− 0.176*** (− 5.08)	− 0.118*** (− 3.46)		− 0.111*** (− 3.66)	− 0.141*** (− 4.17)		− 0.119*** (− 4.20)	− 0.0895** (− 1.98)		− 0.0854** (− 2.57)
Gini, lag		− 0.107*** (− 2.92)	− 0.0756* (− 1.93)		− 0.0553 (− 1.44)	− 0.0524* (− 1.72)		− 0.0908*** (− 3.30)	− 0.0925*** (− 3.45)		− 0.00339 (− 0.08)	− 0.0356 (− 1.10)
Hansen (*p* value)	0.210	0.160	0.290	0.253	0.242	0.491	0.217	0.295	0.493	0.134	0.269	0.470
*Poverty gap, P1, poverty line = US$ 4.0*												
P1, lag	− 0.169*** (− 3.57)		− 0.183*** (− 5.61)	− 0.125*** (− 3.41)		− 0.120*** (− 4.23)	− 0.0993*** (− 3.76)		− 0.104*** (− 4.71)	− 0.0706* (− 1.86)		− 0.0709** (− 2.33)
Gini, lag		− 0.107*** (− 2.92)	− 0.0871** (− 2.03)		− 0.0553 (− 1.44)	− 0.0451 (− 1.15)		− 0.0908*** (− 3.30)	− 0.102*** (− 3.78)		− 0.00339 (− 0.08)	− 0.0326 (− 1.08)
Hansen (*p* value)	0.0985	0.160	0.267	0.208	0.242	0.581	0.208	0.295	0.683	0.191	0.269	0.540
*Squared poverty gap, P2, poverty line = US$ 1.25*												
P2, lag	− 0.413*** (− 4.15)		− 0.347*** (− 4.18)	− 0.197*** (− 2.85)		− 0.177** (− 2.36)	− 0.300*** (− 4.54)		− 0.237*** (− 4.22)	− 0.230** (− 2.35)		− 0.197** (− 2.56)
Gini, lag		− 0.107*** (− 2.92)	− 0.0518 (− 1.07)		− 0.0553 (− 1.44)	− 0.0370 (− 1.15)		− 0.0908*** (− 3.30)	− 0.0709** (− 2.52)		− 0.00339 (− 0.08)	− 0.00956 (− 0.30)
Hansen (*p* value)	0.431	0.160	0.420	0.172	0.242	0.567	0.212	0.295	0.528	0.153	0.269	0.662
*Squared poverty gap, P2, poverty line = US$ 2.0*												
P2, lag	− 0.266*** (− 3.70)		− 0.239*** (− 4.81)	− 0.150*** (− 3.63)		− 0.149*** (− 3.77)	− 0.203*** (− 4.90)		− 0.164*** (− 4.45)	− 0.139** (− 2.19)		− 0.116** (− 2.41)
Gini, lag		− 0.107*** (− 2.92)	− 0.0652* (− 1.68)		− 0.0553 (− 1.44)	− 0.0442 (− 1.31)		− 0.0908*** (− 3.30)	− 0.0820*** (− 2.79)		− 0.00339 (− 0.08)	− 0.0241 (− 0.81)
Hansen (*p* value)	0.270	0.160	0.318	0.210	0.242	0.587	0.211	0.295	0.521	0.127	0.269	0.622
*Squared poverty gap, P2, poverty line = US$ 4.0*												
P2, lag	− 0.201*** (− 3.58)		− 0.196*** (− 5.44)	− 0.131*** (− 3.06)		− 0.127*** (− 3.78)	− 0.132*** (− 3.98)		− 0.123*** (− 4.59)	− 0.0850* (− 1.85)		− 0.0833** (− 2.26)
Gini, lag		− 0.107*** (− 2.92)	− 0.0801** (− 2.03)		− 0.0553 (− 1.44)	− 0.0493 (− 1.53)		− 0.0908*** (− 3.30)	− 0.0980*** (− 3.89)		− 0.00339 (− 0.08)	− 0.0327 (− 1.55)
Hansen (*p* value)	0.136	0.160	0.285	0.248	0.242	0.499	0.188	0.295	0.487	0.160	0.269	0.603

See note in Table 4

### Alternative poverty data

As explained in Sect. 3.1, our use of lognormal-based poverty data is driven by the intent to achieve sample coverage as large as possible. However, one may wonder if that choice has a significant effect on our empirical results. To address this concern, we next re-compute our system GMM estimates using only the PovcalNet poverty data. However, as already noted, the small size (especially in the time dimension) of the raw PovcalNet sample would pose a major obstacle to our estimation approach. Thus, to expand the sample size, we use the interpolated poverty series provided in PovcalNet, as discussed in Sect. 3.1. The main difference between our lognormal poverty measures and those from PovcalNet is not the lognormal approximation, but the reference average income used. In PovcalNet, poverty is directly computed from the income distribution of the household surveys, hence the reference point is the mean level of household income obtained from the survey. In our lognormal approach, average income is given by the 2005 PPP-adjusted GDP per capita from the national accounts. We already discussed the advantages of using this approach in Sect. 3.1.

The poverty headcount values from the interpolated PovcalNet series are fairly similar to those from our constructed P0 with a US$ 2 poverty line (see Table 1 above): While the PovcalNet poverty median is higher (6.8%), the sample average and standard deviation (19% and 24%, respectively), and the minimum and maximum values are similar to those in our baseline data. Moreover, the two poverty series are closely correlated: Over the common sample, the correlation is 0.89 (see Table 14). Further inspection reveals that the correlation is higher for the more recent data, reaching 0.93 in 2005 and 0.96 in 2010.

Table 8 shows estimation results for models M1, M2, M3 and M4 using the PovcalNet interpolated poverty series and our preferred system GMM specification. Comparison with Table 4 reveals that the results are robust to the use of this alternative source of poverty data: Poverty consistently carries a negative coefficient, significant in all cases but one. In turn, the coefficient on inequality is also negative in most instances, but insignificant in three out of eight cases.

**Table 8:** System GMM estimates: robustness to the use of PovcalNet data

	M1. Skeleton model			M2. Extended with education and inv. prices			M3. Extended with policy variables			M4. Extended with policy and infrastructures		
P0, lag	− 0.0959*** (0.0244)		− 0.103*** (0.0325)	− 0.0675** (0.0272)		− 0.0537* (0.0323)	− 0.0637** (0.0285)		− 0.0635** (0.0323)	− 0.0327* (0.0183)		− 0.0206 (0.0448)
Gini, lag		− 0.0794* (0.0407)	− 0.0271 (0.0571)		− 0.0783** (0.0366)	− 0.0777** (0.0383)		− 0.105*** (0.0363)	− 0.0865** (0.0412)		0.0218 (0.0491)	0.000979 (0.0694)
log *y*, lag	− 0.0134*** (0.00496)	− 0.000522 (0.00322)	− 0.0166*** (0.00498)	− 0.00978** (0.00465)	0.00155 (0.00333)	− 0.00822* (0.00498)	− 0.0141** (0.00594)	− 0.00678** (0.00290)	− 0.0160*** (0.00576)	− 0.0246** (0.0103)	− 0.0289** (0.0123)	− 0.0351*** (0.0121)
Inv. deflator, lag				− 0.0256*** (0.00749)	− 0.0355*** (0.0119)	− 0.0270*** (0.00792)						
Female educ., lag				0.000286 (0.00584)	0.00111 (0.00629)	0.000747 (0.00695)				0.000651 (0.00376)	0.00286 (0.00440)	0.00464 (0.00476)
Male educ., lag				0.00145 (0.00634)	− 0.00156 (0.00668)	− 0.0000402 (0.00700)						
Inflation							− 0.000204 (0.000193)	0.000531* (0.000297)	0.000430 (0.000353)	− 0.000114 (0.000248)	0.000823 (0.000520)	0.000887 (0.000602)
Trade openness (log)							0.0284** (0.0117)	0.0320** (0.0144)	0.0280* (0.0144)			
Gov. size (log)							− 0.00351 (0.0125)	0.00171 (0.0102)	0.00139 (0.0118)			
Infrastructure, lag										0.0208*** (0.00773)	0.0284*** (0.0105)	0.0286* (0.0151)
m2-test (*p* value)	0.125	0.345	0.140	0.00886	0.0692	0.0129	0.115	0.990	0.383	0.0668	0.219	0.109
AR(3) (*p* value)	0.0812	0.231	0.383	0.152	0.255	0.475	0.247	0.478	0.470	0.136	0.0734	0.466
Hansen (*p* value)	0.0304	0.102	0.0159	0.0934	0.153	0.173	0.114	0.318	0.206	0.246	0.182	0.196
Diff-Hansen for levels (*p* value)	0.422	0.459	0.087	0.710	0.586	0.556	0.432	0.932	0.816	0.618	0.317	0.205
Num. obs	522	522	522	474	474	474	491	491	491	360	360	360
Num. countries	136	136	136	116	116	116	130	130	130	81	81	81
Num. instruments	32	43	48	102	100	96	102	97	96	80	67	68

See note in Table 5. From PovcalNet, the poverty line is 1.90 US$ 2011, which updates the previous line of 1.25 US$ 2005 (Ferreira et al. 2016). We use the interpolated poverty series provided in PovcalNet, which start in 1981 and are reported every 3 years. To construct a non-overlapping 5-years panel data similar to the one used in our baseline specification, and match poverty data with all other variables (growth and other controls), we use a “closest” criteria or take the average if two poverty observations are 1 year above and one below the assigned year

### Additional controls

Next, we assess the robustness of our results to the use of alternative controls. We focus on two extensions. First, we consider alternative measures of education to proxy for human capital. Second, we consider a set of institutional quality variables. Results are shown in Table 19 in the Appendix 6.

In model M2, we added male and female education separately, following Perotti (1996) and Owen et al. (2002). Here, we estimate several variants of model M2, using average years of schooling, on the one hand, and the percentage of the population with at least primary or secondary education, on the other hand (first and second columns in Table 19).

In turn, we consider two of the most widely used measures of the quality of institutions (see also Table 13 in Appendix 2): an index of democratic accountability (“democracy”), and an index of government stability (“stability”), information taken from the political risk module of the International Country Risk Database.^22^ Columns 3, 4 and 5 of Table 19 extend models M2, M3 and M4 with these institutional variables; column 6 reports the estimation results when jointly including all the variables from M2, M3 and M4.

Finally, and just for illustrative purposes, we report (in the last column of the table) estimates of a model including all the controls. They should be taken with caution, however, given the sharp reduction in sample size (by almost half relative to columns 1–2) and the high degree of collinearity among the regressors.

Estimated coefficients for the percentage of population with primary and secondary education are positive and significant. In the extended specifications with institutional variables, the coefficients of both the quality of democracy and government stability are positive and, in most cases, significant, confirming that the quality of institutions is positively correlated with growth. More importantly, the baseline estimation results for poverty (consistently negative) and inequality (its sign and significance depends on the particular specification) are robust to the inclusion of all these additional controls.

### Alternative econometric specifications

We also performed a number of other robustness checks concerning the empirical specification and estimation approach. To save space, we just provide a brief summary here (results are available upon request). First, we modified the system GMM estimation employing different lag structures—e.g., using *y*_*it−s*_, *p*_*it−s*_, *g*_*it−s*_ and *x*_*it−s*_, for *s* ≥ 4 for the first-difference equation and Δ*y*_*it−*4_, Δ*p*_*it−*4_, Δ*g*_*it−*4_ and Δ*x*_*it−*4_ for the level equation—or using 1-step instead of 2-step estimates. We also experimented with a modified version of the basic empirical equation including a quadratic term in the Gini coefficient. The main conclusion is that the significantly negative effect of poverty on growth is quite robust to all these variations in specification and estimation approach, while the inequality-growth relationship is highly fragile.

Finally, we also re-estimated the models in a pure cross section of countries, with the variables expressed as averages over the entire sample period, capturing what could be viewed as the long-run relationship between them. The estimated poverty coefficient remains uniformly negative and significant, although its precision declines somewhat relative to the panel estimates. In turn, inequality tends to show a negative and significant coefficient, more frequently than in the panel estimates, consistent with recent evidence (e.g., Halter et al 2014; Berg et al. 2018) that inequality exerts a negative long-run impact on growth.

## Poverty regimes

### The effect of poverty and inequality on growth

The nonparametric analysis in the preceding section hinted at possible nonlinear effects of poverty and inequality on growth. To take a deeper look, we estimate alternative versions of Eqs. (1)–(3) allowing for different coefficients on lagged poverty and lagged inequality depending on whether the lagged value of P0 lies above or below the sample median (2.7% for our baseline P0, see Table 1). We follow the same strategy conditioning instead on the lagged level of inequality, and estimate Eqs. (1)–(3) allowing for different coefficients on poverty and inequality depending on whether the lagged Gini coefficient lies above or below its sample median (39.8%, see Table 1). Table 9 reports estimates distinguishing whether poverty is above or below the median—what we shall label the ‘high poverty regime’ and ‘low poverty regime,’ respectively. In turn, Table 10 reports the estimates distinguishing whether inequality is above or below the median—the ‘high inequality regime’ and ‘low inequality regime,’ respectively. In both cases, we use the baseline system GMM specification (Table 5).

**Table 9:** Estimation results by poverty regimes: baseline system GMM

	M1. Skeleton model			M2. Extended with education and inv. prices			M3. Extended with policy variables			M4. Extended with policy and infrastructures			
P0, lag (P0 ≤ *Median*)	0.427 (0.49)		− 0.101 (− 0.16)	− 0.729 (− 0.92)		− 0.944 (− 1.29)	− 0.293 (− 0.44)		− 0.430 (− 0.61)	1.230 (1.44)		− 0.320 (− 0.41)	− 1.019 (− 0.84)
P0, lag (P0 > *Median*)	− 0.125*** (− 4.30)		− 0.124*** (− 5.04)	− 0.0881*** (− 4.08)		− 0.0928*** (− 5.25)	− 0.0733*** (− 3.25)		− 0.0784*** (− 3.65)	− 0.0868*** (− 2.84)		− 0.0586*** (− 2.66)	− 0.0927** (− 2.31)
Gini, lag (P0 ≤ *Median*)		− 0.0553 (− 0.81)	− 0.0589 (− 1.24)		− 0.00103 (− 0.02)	− 0.00504 (− 0.10)		− 0.0689 (− 1.52)	− 0.0759 (− 1.50)		− 0.0285 (− 0.46)	− 0.0224 (− 0.55)	− 0.0114 (− 0.14)
Gini, lag (P0 > *Median*)		− 0.138*** (− 2.83)	− 0.0980*** (− 2.63)		− 0.0544 (− 1.23)	− 0.0547 (− 1.46)		− 0.0969*** (− 2.85)	− 0.107*** (− 3.17)		− 0.0546 (− 1.00)	− 0.0409 (− 1.40)	− 0.0759 (− 0.95)
log *y*, lag	− 0.0208*** (− 4.56)	− 0.0153*** (− 2.73)	− 0.0275*** (− 5.31)	− 0.0160*** (− 4.45)	− 0.0115* (− 1.89)	− 0.0236*** (− 6.43)	− 0.0179*** (− 4.86)	− 0.0134*** (− 2.73)	− 0.0243*** (− 5.32)	− 0.0420*** (− 2.98)	− 0.0374*** (− 3.29)	− 0.0344*** (− 4.65)	− 0.0504*** (− 6.72)
m2 (*p* value)	0.097	0.294	0.205	0.106	0.138	0.168	0.379	0.527	0.539	0.195	0.456	0.389	0.652
Hansen (*p* value)	0.0990	0.0476	0.256	0.211	0.111	0.354	0.408	0.238	0.476	0.163	0.425	0.990	0.122
Num. obs	745	745	745	676	676	676	655	655	655	477	477	477	477
Num. countries	156	156	156	131	131	131	147	147	147	88	88	88	88
Num. Instruments	55	55	85	100	100	130	97	97	127	77	77	127	41

Baseline system GMM estimates: 1 lag in the instrument matrix, starting at *t* − 3. See also the note to Table 4. In the last column of the table, to further reduce the number of instruments in model M4, we consider the case with 2 lags, starting at *t* − 3, and using the collapse option. The sample is divided according with the sample median of P0, which is 2.7% for our baseline P0 with poverty line of 2US$

**Table 10:** Estimation results by inequality regimes: baseline system GMM

	M1. Skeleton model			M2. Extended with education and inv. prices			M3. Extended with policy variables			M4. Extended with policy and infrastructures			
P0, lag (Gini ≤ *Median*)	− 0.109*** (− 3.33)		− 0.0957*** (− 4.04)	− 0.0789*** (− 4.54)		− 0.0924*** (− 4.64)	− 0.0561** (− 2.51)		− 0.0681*** (− 3.09)	− 0.0529** (− 2.06)		− 0.0527* (− 1.68)	− 0.0700** (− 1.97)
P0, lag (Gini > *Median*)	− 0.146*** (− 4.87)		− 0.115*** (− 3.93)	− 0.0976*** (− 4.15)		− 0.0696*** (− 2.93)	− 0.105*** (− 4.03)		− 0.0821*** (− 3.64)	− 0.102*** (− 3.50)		− 0.0530** (− 2.13)	− 0.0935** (− 2.57)
Gini, lag (Gini ≤ *Median*)		0.0343 (0.32)	− 0.0374 (− 0.48)		0.119* (1.86)	0.0885 (1.60)		− 0.0408 (− 0.52)	− 0.0461 (− 0.73)		0.0405 (0.36)	0.00487 (0.08)	0.0931 (0.71)
Gini, lag (Gini > *Median*)		− 0.0397 (− 0.53)	− 0.0484 (− 0.91)		0.0345 (0.71)	0.0223 (0.55)		− 0.0683 (− 1.22)	− 0.0647 (− 1.51)		− 0.00858 (− 0.10)	− 0.00988 (− 0.22)	0.0139 (0.17)
log *y*, lag	− 0.0230*** (− 5.98)	− 0.00713*** (− 2.91)	− 0.0203*** (− 6.25)	− 0.0152*** (− 5.05)	− 0.00585* (− 1.82)	− 0.0163*** (− 5.55)	− 0.0186*** (− 4.63)	− 0.00851*** (− 3.26)	− 0.0190*** (− 6.22)	− 0.0262*** (− 2.73)	− 0.0301*** (− 3.68)	− 0.0353*** (− 4.53)	− 0.0368*** (− 3.86)
m2 (*p* value)	0.1000	0.130	0.142	0.0681	0.0628	0.0770	0.352	0.475	0.441	0.203	0.359	0.277	0.285
Hansen (*p* value)	0.116	0.0375	0.168	0.130	0.0793	0.430	0.242	0.137	0.377	0.0907	0.232	0.988	0.254
Num. obs	745	745	745	676	676	676	655	655	655	477	477	477	477
Num. countries	156	156	156	131	131	131	147	147	147	88	88	88	88
Num. Instruments	55	55	85	100	100	130	97	97	127	77	77	127	41

Baseline system GMM estimates: 1 lag in the instrument matrix, starting at *t* − 3. See also the note to Table 4. In the last column of the table, to further reduce the number of instruments in model M4, we consider the case with 2 lags, starting at *t* − 3, and using the collapse option. The sample is divided according with the sample median of the Gini index, which is 39.8%

Table 9 shows that, under the low poverty regime, the impact of poverty on growth is negative but statistically insignificant. However, it is negative and highly significant under the high poverty regime. In turn, the estimated coefficient on the Gini index is in most cases negative, but it turns significant only for high poverty rates and for the M1 and M3 model specifications. Thus, like with the unconditional estimates, while the result for poverty is robust, the result for inequality is not. In contrast, Table 10 shows that, when we condition on the lagged level of inequality, the estimated coefficients on poverty and inequality exhibit very little variation across inequality regimes. In effect, they are very similar to the unconditional estimates from Table 5.

As a final exercise, we allow the growth effects of poverty and inequality to vary across countries according to their level of development. Specifically, we divide the sample countries into two groups, developed and developing, with the distinction drawn according to the World Bank classification. The “developed” group comprises countries classified as upper-middle income, high-income non-OECD, and high-income OCDE; the “developing” group comprises those classified as low-income and lower-middle income. The estimation results, reported in Table 11, are similar to those obtained in Table 9 when the two groups are drawn according to the median headcount poverty rate: The negative impact of poverty on growth is larger and more significant for developing countries than for developed ones; indeed, for the latter, the effect is insignificant in most cases. The same applies to the coefficient estimate of the Gini index.

**Table 11:** Estimation results: developed vs developing countries: baseline system GMM

	M1. Skeleton model			M2. Extended with education and inv. prices			M3. Extended with policy variables			M4. Extended with policy and infrastructures			
													( +)
P0, lag (developing)	− 0.121*** (− 4.78)		− 0.0919*** (− 4.37)	− 0.0870*** (− 4.04)		− 0.0583*** (− 2.88)	− 0.0975*** (− 4.36)		− 0.0792*** (− 4.57)	− 0.0880*** (− 3.27)		− 0.0461** (− 2.20)	− 0.0712* (− 1.73)
P0, lag (developed)	− 0.0563** (− 2.19)		− 0.0735*** (− 3.11)	− 0.0199 (− 0.77)		− 0.0578*** (− 2.65)	0.00886 (0.27)		− 0.0313 (− 1.24)	0.0383 (0.83)		0.0383 (0.65)	− 0.0121 (− 0.14)
Gini, lag (developing)		− 0.243*** (− 3.71)	− 0.152** (− 2.40)		− 0.177*** (− 3.46)	− 0.107** (− 2.28)		− 0.198*** (− 3.63)	− 0.129*** (− 3.08)		− 0.161** (− 2.46)	− 0.0301 (− 0.66)	− 0.00693 (− 0.08)
Gini, lag (developed)		− 0.104* (− 1.79)	− 0.0834** (− 2.03)		− 0.0506 (− 1.19)	− 0.0349 (− 1.03)		− 0.0988*** (− 2.69)	− 0.0832*** (− 2.94)		− 0.0521 (− 0.78)	− 0.0149 (− 0.56)	0.0427 (0.69)
log *y*, lag	− 0.0196*** (− 5.68)	− 0.0210*** (− 4.02)	− 0.0269*** (− 4.97)	− 0.0151*** (− 4.62)	− 0.0215*** (− 4.46)	− 0.0217*** (− 5.23)	− 0.0185*** (− 5.96)	− 0.0188*** (− 4.81)	− 0.0230*** (− 6.95)	− 0.0344*** (− 3.34)	− 0.0385* (− 1.96)	− 0.0310*** (− 3.48)	− 0.0502*** (− 5.12)
m2 (*p* value)	0.138	0.372	0.270	0.101	0.202	0.119	0.459	0.541	0.524	0.409	0.502	0.307	0.307
Hansen (*p* value)	0.0986	0.128	0.535	0.139	0.145	0.540	0.148	0.238	0.655	0.164	0.276	0.997	0.192
Num. obs	745	745	745	676	676	676	655	655	655	477	477	477	477
Num. countries	156	156	156	131	131	131	147	147	147	88	88	88	88
Num. instruments	55	55	85	100	100	130	97	97	127	77	77	127	41

Baseline system GMM estimates: 1 lag in the instrument matrix, starting at *t* − 3. See also the note to Table 4. In the last column of the table, to further reduce the number of instruments in model M4, we consider the case with 2 lags, starting at *t* − 3, and using the collapse option. Country groups are drawn according to the degree of development following the World Bank classification. Developed group: upper-middle income, high-income non-OECD, and high-income OCDE countries; Developing group: low-income and lower-middle income countries

### The indirect effects of inequality on growth

The coefficient $\varphi_{1}$ in (3) reflects the direct effect of inequality on growth, for given lagged poverty and per capita income levels. However, the overall impact of inequality on growth also depends on how inequality affects poverty. Thus, from (3),^23^

4 $$\partial \left( {\ln y_{it} - \ln y_{it - 1} } \right)/\partial g_{it - 1} = \varphi_{1} + \delta_{1} \left( {\frac{{\partial p_{it - 1} }}{{\partial g_{it - 1} }}} \right).$$

We next examine the indirect effect of inequality on growth, as defined by the second term in the right-hand side of (4), across alternative regimes. Specifically, we only consider the values of $\delta_{1}$ from different poverty regimes (Table 9) because, as Table 10 shows, conditioning on high and low inequality yields estimates of $\delta_{1}$ very similar to the unconditional ones. In the same spirit, to evaluate $\partial p/\partial g$ in (4), we estimate the following equation:

5 $$p_{it} = a_{i} + d_{t} + b \cdot \ln y_{it} + c \cdot g_{it} + \eta_{it} ,$$

both for the entire sample and splitting the sample in two depending on whether poverty is above or below the median.

We estimate (5) using the WG estimator. To take care of the potential bias arising from simultaneity between poverty and income, we instrument the log of income in (5) with past values of the saving rate (Acemoglu et al 2008). Results are shown in Table 12: The left panel reports the within-group estimates and the right panel reports the instrumental variable (IV) estimates. In both cases, we include time dummies and country fixed effects in the regressions. For the IV estimation, we report the first-stage Kleibergen–Paap *F* statistic to test for the weakness of our set of instruments. In all cases, the test statistic is above the Stock and Yogo (2005) critical values. Thus, we can reject the null of weak instruments. The *p* value of Hansen’s J test of over-identification exceeds 0.1 in all cases, suggesting that we cannot reject the hypothesis that the instruments are valid. Both estimation strategies lead to similar conclusions. The poverty-inequality slope is positive but relatively flat for low poverty rates (i.e., when P0 is below the sample median), and strongly positive for high poverty rates (when P0 is above the sample median). The implication is that inequality changes have a strong effect on poverty when poverty is high, but not when it is low.

**Table 12:** The effect of income and inequality on poverty

	Within-group estimates			Instrumental variable		
	Entire sample	P0 < Median	P0 > Median	Entire sample	P0 < Median	P0 > Median
log *Y*	− 0.160*** (− 4.21)	− 0.00301* (− 1.83)	− 0.380*** (− 16.32)	− 0.150*** (− 5.12)	− 0.00619** (− 2.46)	− 0.374*** (− 13.60)
Gini	0.368*** (3.64)	0.0244*** (4.18)	0.766*** (11.01)	0.307*** (4.17)	0.0253*** (4.15)	0.725*** (10.62)
Country FE	Yes	Yes	Yes	Yes	Yes	Yes
Year FE	Yes	Yes	Yes	Yes	Yes	Yes
Num. observations	803	402	401	566	284	282
R2	0.434	0.296	0.867	0.396	0.181	0.834
Kleibergen-Paap *F*-stat				44.67	24.71	17.87
Under-identification *F*-stat				56.37	21.32	28.88
(P value)				0.000	0.000	0.000
Hansen overidentifying				0.514	0.0775	0.180
(*p* value)				0.473	0.781	0.671

The dependent variable is the headcount poverty rate (for US$ 2 as poverty line). The sample is divided according with the sample median of this baseline P0, which is 2.7%. The left panel shows WG estimates, including both country and year fixed effects. The IV approach attempts to overcome the potential double causality bias between poverty and income. We use 2 lags of the saving rate as instrument for the log of income (Acemoglu et al. 2008). We show results of three tests. First, a test *F* of under-identification (the null hypothesis is “under-identification”). Second, the Kleiberger-Paap *F*-stat (the null hypothesis is that instruments are not weak), using the reference values in Stock and Yogo (2005): A rule of thumb to not reject the null is that the *F*-teat is above 10. The Hansen overidentifying test (the null hypothesis is that the instruments are exogenous). Robust *t* statistics in parentheses: *** denotes significance at 1%, ** at 5%, * at 10%

**Fig. 3:**
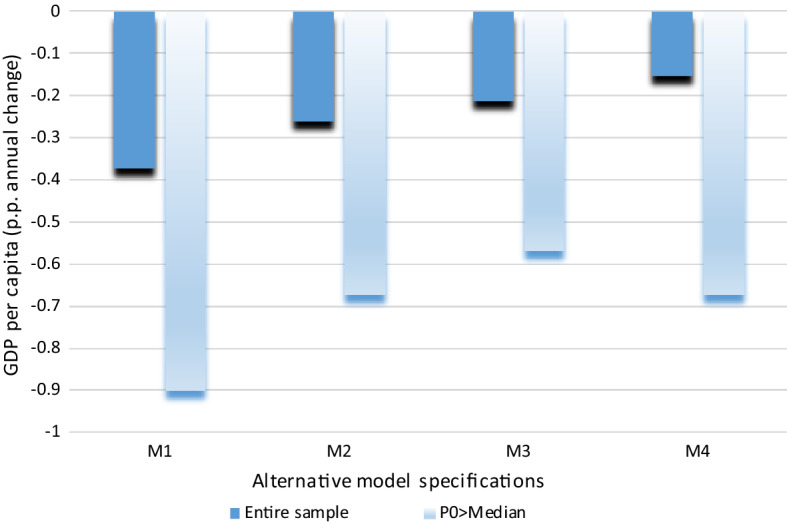
Inequality and growth under different poverty regimes. Indirect effect on growth of a 1-standard deviation (0.10) increase in the Gini coefficient from its median (0.40). *Note* The indirect effect is given by the second term in (4). For the entire sample, we use $$\delta_{1}$$ from Table 4, along with the poverty-inequality coefficient in Table 12 (the IV estimates). For the case of P0 > Median, we use $$\delta_{1}$$ from Table 9. for P0 > Median, along with the corresponding poverty-inequality coefficient from Table 12 (for the IV approach). The sample is divided according with the sample median of P0, which is 2.7% for our baseline P0 with poverty line of 2US$. M1, M2, M3 and M4 denote the alternative sets of control variables included in (1)–(3)

**Fig. 4:**
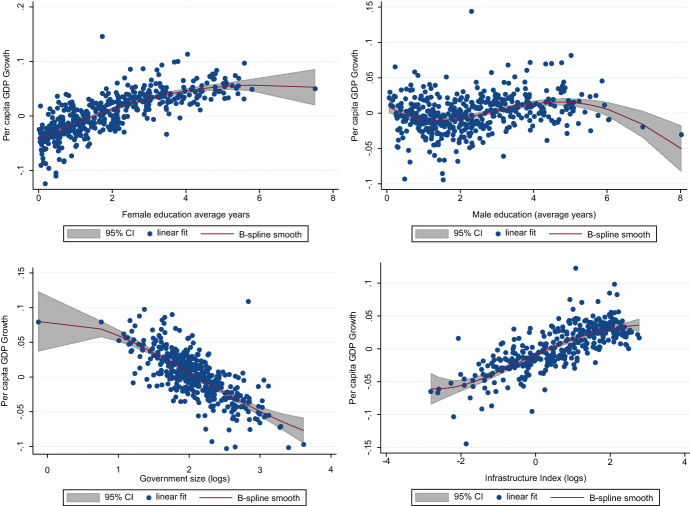
Per capita GDP growth and several key controls (nonparametric estimates). *Note* Estimations made using the Baltagi and Li’s (2002) semiparametric fixed-effects regression estimator. We estimate Eq. (3) for model M2, M3 and M4 allowing for a nonparametric (approximated by a spline interpolation) specification for one particular regressor at a time. In this example, we show the cases of male and female education (model M2), government size (model M3) and the Infrastructure index (model M4)

To illustrate numerically the indirect effect of inequality on growth, we combine these estimates of $\partial p/\partial g$ with the estimates of $\delta_{1}$ from Table 9. Since when P0 is below the median the estimate of $\delta_{1}$ is statistically insignificant, the indirect effect of inequality on growth is negligible in this situation. Thus, we focus on the high poverty subsample. Figure 3 shows the estimated indirect effect of inequality on growth (expressed in percent per year) for models M1, M2, M3 and M4, and two alternative sets of estimates: the unconditional estimates, ignoring the prevailing poverty regime, and the estimates obtained when poverty is above the median. Specifically, the figure illustrates the consequences of a one-standard deviation increase in the Gini coefficient (i.e., by 0.10 according to Table 1). In all cases, we use estimation results from Eq. (3).^24^

To compute the unconditional indirect effect of inequality on growth, we combine the estimated $\partial p/\partial g$ from Table 12 for the IV case (equal to 0.307) with the unconditional estimates of $\delta_{1}$ in (3) from the baseline system GMM (Table 5). The indirect effect of inequality (through poverty) on growth is always negative. Specifically, a 10-point increase in the Gini coefficient generates, through the indirect effect, a decrease in annual growth by about 0.20 (model M4) to 0.40 (model M1) percentage points. For the high poverty case (P0 above the median), we employ the estimates of $\delta_{1}$ applicable to that regime from Table 9, and the estimated $\partial p/\partial g$ of 0.725 from Table 12 for the IV approach. In this scenario, the indirect impact of inequality on growth is uniformly negative, and larger than the unconditional one, i.e., a 10-point increase in the Gini coefficient reduces growth on average by 0.6 percentage points (model M3) or 0.9 percentage points (model M1) per year.

## Conclusions

This paper has examined two issues that have received limited attention in the otherwise extensive empirical literature of growth, inequality and poverty. First, the paper provides an empirical assessment of the impact of poverty on growth. Second, the paper also highlights the indirect effect of inequality on growth accruing through poverty.

The paper uses a large panel dataset including 804 observations covering 158 countries and spanning the years 1960–2010. The empirical strategy involves including inequality and poverty indicators among the explanatory variables in an otherwise standard empirical growth equation. On the whole, the results reveal a consistently negative and strongly significant correlation of poverty with subsequent growth. Its magnitude is economically significant too: A 10 percentage point decrease in the headcount poverty rate is associated with a rise in annual per capita real growth of 0.5% to 1.2%. However, further analysis reveals that the significance of the effect depends on the prevailing level of poverty. Specifically, when the level of poverty is low (below the sample median), the growth effect of poverty is not statistically significant. In contrast, when the level of poverty is high, changes in the poverty headcount rate do show a significantly negative association with subsequent growth.

In contrast, we find that the link between inequality and growth is fragile. It can take either sign depending on the particular model and econometric approach employed. Consistent with previous results in the literature, we find a positive (significant in some specifications) sign when using the within dimension of the data, and a negative one (also significant at times) when using the cross-country dimension. Still, the indirect effect of inequality (through poverty) on growth is robustly negative, especially when the level of poverty is above the sample median. Its magnitude is also economically significant, e.g., a 10-percentage point decrease in the Gini coefficient is associated with an increase in per capita growth ranging between 0.2% and 0.4% in the full sample, and over twice as large in the above-median poverty subsample.

More broadly, our findings underscore the potential growth cost of adverse shocks to poverty, triggered by events such as drops in income or surges in inequality. Because poverty deters growth, a shock that causes poverty to rise may lead to a subsequent growth slowdown. The COVID-19 pandemic provides a relevant example. The growth collapse triggered by the pandemic is estimated to have raised extreme poverty in developing countries by some 120 million individuals in 2020 (or, equivalently, more than 20% over the pre-pandemic trend), with an even larger increase expected for 2021 (Lakner et al. 2021). However, these figures are biased downward because they assume no change in inequality, and the evidence shows that pandemics typically raise inequality (Furceri et al. 2020). Rising inequality adds indirectly to the poverty surge. In fact, while reliable data are not yet available, some rough estimates (e.g., IMF 2020) suggest that the COVID-19 pandemic is leading to a substantial increase in inequality in emerging and developing countries, especially poorer ones, which will result in a further increase in poverty rates and a major setback to the fight against global poverty. Our results imply that, in addition, the poverty rise may act as a drag on future growth, potentially triggering a vicious circle of stagnating incomes and rising poverty.

From the policy perspective, the finding that poverty tends to deter growth has potentially major implications. Supporting the incomes of poor households in the face of adverse shocks—such as COVID-19—through expanded social assistance (e.g., cash transfers, food stamps and in-kind nutrition) and enhanced social protection (e.g., relaxing eligibility criteria for unemployment insurance, expanding sick pay), as well as improved access to education and health care, can help contain the impact on aggregate poverty and the knock-on effect on growth. More broadly, our results suggest that these kinds of policies may be indicated not only for reasons of social equity and fairness, but also from the point of view of overall growth and prosperity.

## Appendix 1: Lognormal approximation of alternative poverty measures

Following Dollar and Kraay (2002), López and Servén (2015) or Pinkovskiy and Sala-i-Martin (2013), we construct a set of poverty figures (the headcount ratio, P0, the poverty gap, P1 and the squared poverty gap, P2) using a lognormal approximation on the basis of the observed per capita income levels and Gini coefficients, which are available much more widely than survey-based poverty data.

The use of the lognormal approximation to the distribution of income dates back to Gibrat (1931). The literature employs also other functional forms, such as the Pareto, the gamma or the Weibull distribution, but the lognormal is the more widely used. Indeed, López and Servén (2006) compare the quintile income shares generated by a lognormal distribution with their observed counterparts using data from over 1000 household surveys and find the lognormal approximation fits the data extremely well, so that they are unable to reject the null hypothesis that per capita income follows a lognormal distribution.

Under lognormality, given the Gini coefficient (*g*), the standard deviation (σ) of the log of income is given by $\sigma = \sqrt {\Phi^{ - 1} \left( {\frac{1 + g}{2}} \right)}$, where $\Phi ( \cdot )$ is the standard normal cumulative distribution function. Using this expression and the log of per capita income (*y*), we can compute the FGT family of poverty measures for a given poverty line *z* as:

$$\begin{array}{*{20}l} {P0 = \Phi \left( {\frac{\log (z) - y}{\sigma } + \frac{\sigma }{2}} \right)} \hfill \\ {P1 = \Phi \left( {\frac{\log (z) - y}{\sigma } + \frac{\sigma }{2}} \right) - \frac{{e^{y} }}{z}\Phi \left( {\frac{\log (z) - y}{\sigma } - \frac{\sigma }{2}} \right)} \hfill \\ {P2 = \Phi \left( {\frac{\log (z) - y}{\sigma } + \frac{\sigma }{2}} \right) - 2\frac{{e^{y} }}{z}\Phi \left( {\frac{\log (z) - y}{\sigma } - \frac{\sigma }{2}} \right) + \left( {\frac{{e^{y} }}{z}} \right)^{2} e^{{\sigma^{2} }} \Phi \left( {\frac{\log (z) - \nu }{\sigma } - \frac{3\sigma }{2}} \right).} \hfill \\ \end{array}$$

## Appendix 2: Data description and cross-correlations

See Tables 13, 14.

**Table 13:** Description of the variables

Name	Description	Source	Num.Obs. (restricted to P0 and Gini sample)	Sample average	Standard deviation
Per capita real GDP	Level of activity and degree of development: PPP Converted GDP Per Capita (Chain Series), at 2005 constant prices	Penn World Tables 7.1	749	9793 US$ (PPP-2005)	10,462 (PPP-2005)
Poverty	The headcount ratio P0 (level of poverty), the poverty gap P1 (intensity), and the squared poverty gap P2 (severity). For the log-logistic measure, the baseline poverty line is US$ 2; for PovcalNet, we use US$ 1.90 as poverty line	Own calculation based on lognormal approximation; PovcalNet	749 (lognormal) 556 (Povcal.)	16.18% (P0) 7.34 (P1) 4.40% (P2) 18.56 (Povcal.)	24.75% (P0) 13.22% (P1) 8.92% (P2) 21.14% (Povcal.)
Gini coefficient	Measure of income inequality (between 0 and 1). Based only on nationally representative surveys (area, population and age), and based on income (net of transfers and taxes) and expenditure figures	UN-WIID2 (2008); PovcalNet	749	40.20%	9.98%
Years of secondary education (total, male and female)	Average years of secondary education of the male population and the average years of secondary education of the female population	Barro and Lee (2013) Educational Attainment Data	684	1.95 (total) 1.77 (female) 2.15 (male)	1.42 (total) 1.44 (female) 1.44 (male)
Attained education (primary and secondary)	Percentage of population (total) with at least primary or secondary education	Barro and Lee (2013) Educational Attainment Data	684	19.5 (primary) 16.2 (secondary)	12.8 (primary) 13.3 (secondary)
Investment prices	Domestic price of investment goods relative to that of the U.S. as a measure of market distortions	Penn World Tables 7.1	745	0.65 (relative to US)	0.31 (relative to US)
Inflation	GDP deflator, as an indicator of macroeconomic stability	World Development Indicators, World Bank	667	16.35%	32.05%
Degree of openness	Volume of trade with respect to its GDP	Penn World Table 7.1	749	75.8%	49.7%
Government size	The ratio of public consumption to GDP: as an indicator of the burden imposed by the government on the economy	Penn World Table 7.1	749	9.65%	5.41%
Infrastruct. Index	Composite index of public infrastructure including: telecommunication sector (number of main telephone lines per 1000 workers), the power sector (the electricity generating capacity in MW per 1000 workers), the transportation sector (the length of the road network—in km. per sq. km. of land area)	World Development Indicators, World Bank. Based on Calderón et al. (2015)	528	0.39	1.33
Democracy	Degree of Democracy: whether there are free and fair elections and the degree of government's accountability. Range of values between 0—minimum democracy—and 6—maximum democracy)	International Country Risk Database	474	4.15	1.46
Government stability	Degree of Government stability: measures the government's ability to carry out its declared program(s) and its ability to stay in office. Range of values between 1—minimum stability—and 12—maximum stability	International Country Risk Database	474	7.71	2.06

**Table 14:** Correlation matrix

	Growth	pcGDP (log)	P0 (US$ 2) lognormal	P0 (Povcalnet)	Gini	Second.yr total	Second.yr female	Second.yr male	Primary attain. (%)
Growth	1.000								
pcGDP(log)	0.152	1.000							
P0 (US$ 2)	− 0.176	− 0.828	1.000						
P0 (Povcal.)	− 0.162	− 0.843	0.892	1.000					
Gini	− 0.184	− 0.484	0.357	0.363	1.000				
Sec.yr total	0.198	0.766	− 0.592	− 0.651	− 0.447	1.000			
Sec.yr female	0.195	0.777	− 0.595	− 0.650	− 0.409	0.989	1.000		
Sec.yr male	0.196	0.738	− 0.575	− 0.638	− 0.477	0.988	0.956	1.000	
Prim.att	0.007	0.202	− 0.154	− 0.193	− 0.088	− 0.151	− 0.146	− 0.150	1.000
Sec.att	0.214	0.641	− 0.529	− 0.579	− 0.404	0.877	0.865	0.871	− 0.184
Inv.Price	− 0.008	0.280	− 0.033	− 0.139	− 0.220	0.238	0.235	0.235	0.012
Inflation	− 0.079	− 0.058	− 0.022	0.000	0.071	− 0.031	− 0.033	− 0.029	− 0.072
Open	0.298	0.143	− 0.168	− 0.192	− 0.087	0.266	0.285	0.240	− 0.120
Gov.Size	− 0.115	− 0.332	0.381	0.453	0.080	− 0.267	− 0.248	− 0.282	− 0.071
Infrast	0.197	0.922	− 0.781	− 0.812	− 0.499	0.741	0.743	0.722	0.218
Democ	0.126	0.682	− 0.443	− 0.516	− 0.377	0.503	0.539	0.456	0.219
Gov.Stab	0.354	0.164	− 0.094	− 0.150	− 0.099	0.225	0.221	0.223	0.020

	Second. attain. (%)	Inv. price (relativ. US)	Inflat	Open, adjust. (log)	Gov. size (log)	Infrast (index)	Democ (0–6 index)	Gov. stab. (0–12 index)
Growth								
pcGDP(log)								
P0 (US$ 2)								
P0 (Povcal.)								
Gini								
Sec.yr total								
Sec.yr female								
Sec.yr male								
Prim.att								
Sec.att	1.000							
Inv.Price	0.173	1.000						
Inflation	− 0.017	− 0.051	1.000					
Open	0.308	− 0.014	− 0.138	1.000				
Gov.Size	− 0.182	− 0.104	− 0.041	− 0.129	1.000			
Infrast	0.657	0.251	− 0.120	0.228	− 0.307	1.000		
Democ	0.432	0.306	− 0.133	0.173	− 0.214	0.704	1.000	
Gov.Stab	0.186	− 0.040	− 0.098	0.267	− 0.110	0.259	0.158	1.000

Variables are transformed in the same way as for the regression analysis. For example, per capita GDP in logs; poverty and the Gini coefficient in levels; openness in logs and adjusted by population, kilometers, to be oil exporters and landlock; government size in logs, etc. We consider two alternative measures of the headcount poverty rate: first, using a lognormal approximation (Dollar and Kraay 2002; Sala-i-Martin 2006; López and Servén 2015), and, for a reduced sample, using the PovcalNet database

## Appendix 3: Alternative system GMM estimation results

See Tables 15, 16.

**Table 15:** Growth, poverty and inequality: system GMM estimates (collapse, all lags)

	M1. Skeleton model			M2. Extended with education and inv. prices			M3. Extended with policy variables			M4. Extended with policy and infrastructures		
P0, lag	− 0.157*** (− 4.14)		− 0.138*** (− 4.06)	− 0.104*** (− 3.85)		− 0.0860*** (− 3.77)	− 0.0963*** (− 4.17)		− 0.0918*** (− 4.61)	− 0.0626** (− 2.06)		− 0.0581** (− 2.08)
Gini, lag		− 0.165** (− 2.53)	− 0.153*** (− 3.01)		− 0.0798* (− 1.77)	− 0.0444		− 0.0289 (− 0.87)	− 0.0766** (− 2.30)		0.0105 (0.32)	− 0.0215 (− 0.60)
						(− 1.05)						
log *y*, lag	− 0.0264*** (− 4.83)	− 0.00454 (− 1.35)	− 0.0271*** (− 5.36)	− 0.0216*** (− 4.22)	− 0.00310 (− 1.18)	− 0.0181*** (− 4.37)	− 0.0172*** (− 5.09)	− 0.0057** (− 2.20)	− 0.0194*** (− 5.80)	− 0.0324*** (− 3.87)	− 0.0174** (− 2.13)	− 0.0303*** (− 3.58)
Inv. deflator, lag				− 0.00251* (− 1.66)	− 0.0032 (− 1.42)	− 0.0021 (− 0.91)						
Female educ., lag				0.00284 (0.60)	0.00890 (1.54)	0.00805 (1.40)				0.0068*** (2.61)	0.0022 (0.59)	0.0059** (2.20)
Male educ., lag				0.00123 (0.27)	− 0.00899 (− 1.35)	− 0.00664 (− 1.03)						
Inflation							0.0008** (2.13)	0.0011*** (2.81)	0.0006 (1.49)	0.0009*** (3.57)	0.0009*** (2.79)	0.0007** (2.14)
Trade openness (log)							0.0202* (1.79)	0.0372*** (4.17)	0.0159* (1.65)			
Gov. size (log)							0.0199 (1.52)	0.00859 (0.88)	0.00910 (0.97)			
Infrastructure, lag										0.0165** (2.32)	0.0175*** (2.61)	0.0143* (1.90)
m2-test (*p* value)	0.147	0.313	0.332	0.0710	0.179	0.115	0.236	0.427	0.397	0.226	0.179	0.262
AR(3) (*p* value)	0.832	0.527	0.575	0.817	0.837	0.955	0.631	0.544	0.532	0.802	0.887	0.804
Hansen (*p* value)	0.00333	0.000907	0.0517	0.0972	0.0428	0.114	0.118	0.0331	0.293	0.266	0.286	0.499
Diff-Hansen, levels (*p* value)	0.135	0.108	0.636	0.463	0.413	0.470	0.746	0.462	0.845	0.569	0.442	0.756
Num. obs	745	745	745	676	676	676	656	656	656	477	477	477
Num. countries	156	156	156	131	131	131	147	147	147	88	88	88
Num. instruments	40	40	55	85	85	100	82	82	97	82	82	97

See Note Table 4. Estimations are done using 2-step system GMM (all lags starting at *t* − 3), but collapsing the matrix of instruments. The instrument set starts at *t* − 3. The difference Hansen test assesses the validity of the instruments for the level equation in system GMM. Robust *t* statistics in parentheses. ***denotes significance at 1%, **at 5%, *at 10%

**Table 16:** Growth, poverty and inequality: system GMM estimates (collapse, reduce)

	M1. Skeleton model			M2. Extended with education and inv. prices			M3. Extended with policy variables			M4. Extended with policy and infrastructures		
P0, lag	− 0.183*** (− 5.21)		− 0.167*** (− 4.98)	− 0.143*** (− 5.55)		− 0.141*** (− 5.96)	− 0.131*** (− 2.82)		− 0.126*** (− 3.59)	− 0.0888*** (− 3.31)		− 0.0849*** (− 3.40)
Gini, lag		− 0.161* (− 1.65)	− 0.0798 (− 0.96)		− 0.136 (− 1.54)	− 0.0411 (− 0.70)		− 0.0575 (− 1.50)	− 0.0972** (− 2.19)		0.0447 (0.86)	− 0.0322 (− 0.91)
log *y*, lag	− 0.0306*** (− 5.34)	0.00134 (0.29)	− 0.0299*** (− 5.03)	− 0.0296*** (− 5.08)	0.00413 (0.78)	− 0.0280*** (− 5.10)	− 0.0255*** (− 3.80)	− 0.00586* (− 1.82)	− 0.0260*** (− 4.04)	− 0.0449***	− 0.0218 (− 1.63)	− 0.0431*** (− 5.02)
										(− 4.50)		
Inv. deflator, lag				0.000917 (0.26)	0.00168 (0.36)	0.00159 (0.39)						
Female educ., lag				0.00563 (0.60)	0.00250 (0.29)	0.00329 (0.37)				0.00757** (2.06)	− 0.00199 (− 0.39)	0.00654* (1.85)
Male educ., lag				− 0.00274 (− 0.30)	− 0.00947 (− 1.00)	− 0.00197 (− 0.22)						
Inflation							0.0009** (1.96)	0.0007 (0.89)	0.0003 (0.34)	0.0013** (2.33)	0.0017* (1.90)	0.0014** (2.44)
Trade openness (log)							0.0199 (1.29)	0.0385*** (3.86)	0.0179 (1.43)			
Gov. size (log)							0.00414 (0.32)	− 0.000404 (− 0.03)	0.00411 (0.35)			
Infrastructure, lag										0.0239** (2.46)	0.0273*** (2.73)	0.0218** (2.52)
m2-test (*p* value)	0.164	0.302	0.266	0.0976	0.258	0.135	0.314	0.469	0.473	0.336	0.160	0.406
AR(3) (*p* value)	0.850	0.508	0.718	0.902	0.888	0.953	0.602	0.592	0.591	0.808	0.876	0.753
Hansen (*p* value)	0.257	0.00672	0.218	0.340	0.0539	0.404	0.298	0.0270	0.631	0.0446	0.105	0.0997
Diff-Hansen, levels (*p* value)	0.360	0.09	0.552	0.424	0.338	0.253	0.618	0.51	0.94	0.173	0.539	0.401
Num. obs	745	745	745	676	676	676	656	656	656	477	477	477
Num. countries	156	156	156	131	131	131	147	147	147	88	88	88
Num. instruments	22	22	28	40	40	46	39	39	45	39	39	45

See note Table 4. Estimations are done using 2-step system GMM (two lags starting at *t* − 3), but collapsing the matrix of instruments. The instrument set starts at *t* − 3. The difference Hansen test assesses the validity of the instruments for the level equation in system GMM. Robust *t* statistics in parentheses. *** denotes significance at 1%, ** at 5%, * at 10%

## Appendix 4: Residual cross-sectional dependence tests

See Table 17.

**Table 17:** Residual cross-sectional dependence tests of main GMM models

	M1		M2		M3		M4	
	CD-stat	P value	CD-stat	P value	CD-stat	P value	CD-stat	P value
First-difference GMM (Table 4)	1.061	0.144	− 2.11	0.017	− 1.001	0.158	− 1.321	0.093
System GMM-baseline (Table 5)	− 1.586	0.0567	− 2.765	0.003	− 0.036	0.486	0.13	0.448
System GMM-collapse all lags (Table 15)	− 2.642	0.004	− 3.421	0	− 1.306	0.096	− 0.951	0.171
System GMM-collapse and reduce (Table 16)	− 3.113	0.001	− 2.976	0.001	− 1.097	0.136	− 0.04	0.484

We show results for the Pesaran’s (2021) CD test and for the model versions including both poverty and inequality. The null hypothesis is the absence of cross-sectional dependence

## Appendix 5: Nonlinearities and nonparametric results

See Table 18 and Fig. 4.

**Table 18:** Growth, poverty and inequality: nonparametric specification for each regressor

Nonparametric	M2			M3			M4		
	Inv. Deflator	Female educ	Male educ	Inflation	Trade open	Gov. Size	Inflation	Female educ	Infrastruc
P0, lag	− 0.0544** (− 2.27)	− 0.0632*** (− 2.64)	− 0.0563** (− 2.34)	− 0.0621*** (− 2.78)	− 0.0654*** (− 2.78)	− 0.0662*** (− 2.80)	− 0.0598** (− 2.18)	− 0.0660** (− 2.24)	− 0.0627** (− 2.01)
Gini, lag	0.0651*** (2.76)	0.0680*** (2.89)	0.0671*** (2.85)	0.0581*** (2.73)	0.0598*** (2.67)	0.0585*** (2.60)	0.0524** (2.10)	0.0517* (1.96)	0.0489* (1.83)
log *y*, lag	− 0.138*** (− 14.56)	− 0.141*** (− 15.17)	− 0.139*** (− 15.08)	− 0.165*** (− 18.74)	− 0.160*** (− 17.43)	− 0.160*** (− 17.28)	− 0.154*** (− 13.88)	− 0.153*** (− 13.10)	− 0.152*** (− 12.66)
N	466	466	466	448	448	448	345	345	345
R2-adj	0.403	0.409	0.407	0.527	0.478	0.478	0.439	0.379	0.385

We estimate Eq. (3) for models M2, M3 and M3 allowing for a nonparametric (approximated by a spline interpolation) specification for one particular regressor at a time (shown in each column of the table). Estimations are based on Baltagi and Li’s (2002) semiparametric fixed-effects regression approach. Each column reports the resulting linear coefficient estimates on inequality, poverty, and lagged income

## Appendix 6: The use of additional controls

Table 19.

**Table 19:** System GMM estimates: robustness to alternative set of controls

	M5	M6	M7	M8	M9	M10	M11
P0, lag	− 0.0909*** (− 4.17)	− 0.0806*** (− 3.38)	− 0.0644** (− 2.43)	− 0.110*** (− 4.05)	− 0.0658*** (− 2.61)	− 0.0516** (− 2.04)	− 0.0388 (− 1.28)
Gini, lag	− 0.0447 (− 1.38)	− 0.0321 (− 1.31)	− 0.106*** (− 2.94)	− 0.0921*** (− 2.67)	− 0.0237 (− 0.59)	0.0102 (0.28)	− 0.0564 (− 1.07)
log *y*, lag	− 0.0183*** (− 5.09)	− 0.0188*** (− 6.20)	− 0.0197*** (− 3.60)	− 0.0260*** (− 4.96)	− 0.0317*** (− 5.02)	− 0.0363*** (− 2.89)	− 0.0300*** (− 2.79)
Inv. deflator, lag	− 0.000744 (− 0.64)	− 0.00133 (− 1.34)	− 0.0169** (− 2.31)			− 0.00830 (− 0.60)	− 0.0204* (− 1.75)
Total sec. yrs educ., lag	0.00201 (0.83)						
Female sec. yrs educ., lag			− 0.00994 (− 1.13)			− 0.00728 (− 0.78)	− 0.0119 (− 1.48)
Male sec. yrs educ., lag			0.0143* (1.70)		0.00619*** (2.88)	0.0110 (1.09)	0.0156* (1.92)
% Secondary attained, lag		0.000427* (1.89)					
% Primary attained, lag		0.000553** (2.30)					
Inflation				0.00132 (1.33)	0.00174** (2.14)	0.00156* (1.73)	0.000450 (0.60)
Trade openness (log)				0.00415 (0.49)		0.0220** (2.11)	0.0226* (1.87)
Gov. size (log)				0.000222 (0.02)		− 0.0233 (− 1.48)	− 0.0196* (− 1.69)
Infrastructure, lag					0.00991 (1.60)	0.0225** (2.07)	0.0128 (1.38)
Democracy			0.00299 (1.04)	0.00463** (2.08)	0.00689*** (2.80)		0.00368 (1.28)
Gov stability			0.00692*** (3.87)	0.00356 (1.60)	0.00576** (2.55)		0.00558*** (3.12)
m2-test (*p* value)	0.0993	0.120	0.0811	0.333	0.354	0.123	0.0671
AR(3) (*p* value)	0.770	0.620	0.951	0.889	0.833	0.850	0.959
Hansen (*p* value)	0.305	0.672	0.688	0.115	0.332	0.107	0.211
Diff-Hansen for levels (*p* value)	0.554	0.822	0.979	0.652	0.610	0.209	0.102
Num. obs	678	678	464	463	360	468	352
Num. countries	131	131	113	118	82	84	78
Num. instruments	120	142	122	86	86	69	78

Estimations are done using 2-step system GMM, starting at *t* − 3, and reducing the number of instruments to: (1) two lags (baseline, see Table 5); (2) one lag (as for model M4 in Table 5); (3) reduce to 3 lags and collapse (as in Table 16 in Appendix 3). In all cases, this different instruments structure is to keep the number of instruments below the number of cross sections to avoid overfitting problems. Notice the purpose of this table is not to compare results between models, but to check for robustness of our main results

## Footnotes

1 The consequences of inequality for poverty are highlighted for example by Bourguignon (2003, 2004) or Ravallion (2005). Marrero and Servén (2018) provide numerical simulations illustrating the magnitude of the effect of inequality on poverty, for given average income.

2 Empirical analyses of the links between aggregate growth, poverty and inequality commonly use an instrumental variable approach. A few papers feature external instruments—e.g., Brueckner et al. (2015), assessing the effect of GDP growth on inequality—but GMM using internal instruments (given by suitably lagged and transformed regressors) is much more commonly used: for example, by Partridge (1997), Forbes (2000), Panizza (2002), or Berg et al. (2018), all of which are concerned with the opposite direction of causality, from inequality to growth.

3 Poverty traps arising from threshold effects have often been offered as a rationale for a ‘big push’ approach to policy. In particular, when large aid programs are coordinated in a multi-faceted way, a ‘big push’ can be effective to engineer growth takeoffs (Banerjee et al., 2015). However, in a cross-country dataset, Easterly (2006) finds that takeoffs are rare and, in general, they are not associated with ‘big push’ strategies.

4 The argument that risk aversion leads to underinvestment goes back to Stiglitz (1969). See also Agenor and Aizenman (2011), who argue that aid volatility could induce poverty traps in poor countries through a similar mechanism.

5 Easterly (2006) investigates (and rejects) a more extreme hypothesis, namely that high poverty countries should show no growth.

6 Erman and te Kaat (2019) show that higher inequality increases growth in physical capital-intensive industries, while it harms grow in industries using skilled labor intensively.

7 A more limited literature has examined the inequality-growth link from the opposite perspective, assessing how income growth affects inequality. Its results are mostly inconclusive, however. For instance, while Brueckner et al. (2015) and Blau (2018) find that GDP growth reduces inequality, Krusell et al. (2000) and Aghion et al. (2019) reach the opposite conclusion.

8 Specifically, we pool the sample and regress the Gini coefficient on a constant, regional dummies and dummy variables indicating whether the survey is stated in terms of gross income or consumption (the omitted category is income net of taxes and transfers). We then subtract the estimated mean difference between these two alternatives and the omitted category to arrive at a set of Gini indices that notionally correspond to the distribution of income net of taxes and transfers. The results of these adjustment regressions are available upon request, but they show similar conclusions as in Dollar and Kraay (2002).

9 The UN-WIID2 Gini index is not always available for the first year of each 5-year interval. In such cases, we allocate the available observation(s) to the closest starting year of a 5-year interval, with a limit of 2 years of difference. When more than one observation is available within the 2-year limit, we take the average. Because of the strong inertia of inequality and poverty time series, using a 1-year limit instead of 2 years, or not using means, yields very similar results (Dollar and Kraay 2002).

10 Our data comprise 121 data points corresponding to 32 low-income countries, 180 to 41 lower-middle income countries, 240 to 44 upper-middle income, 57 to 11 high-income non-OECD, and 206 to 30 high-income OCDE countries. The sample includes 18 observations (2 countries) from North America, 248 (48 countries) from Europe and Central Asia, 159 (28 countries) from Latin American and the Caribbean, 53 (12 countries) from Middle East and North Africa, 144 (40 countries) from Sub-Saharan Africa, 56 (9 countries) from South Asia and 126 (19 countries) from East Asia and the Pacific.

11 This sample size would be too small for many of our exercises, and thus for the robustness tests using PovcalNet data reported in Section V below, we resort to the interpolated PovcalNet series, which allows increasing the sample size to 556 observations. These interpolated information start in 1981 and are reported every three years. Thus, to construct a non-overlapping 5-year panel data similar to the one used in our baseline specification and match the timing of poverty data with that of the other variables (growth and other controls), we use a “closest” criterion or take the average if two poverty observations are one year above and one below the assigned year. We should also note that the current PovcalNet series uses a poverty line of 1.90 2011 US$, which replaces its previous line of 1.25 2005 US$ (see Ferreira et al. 2016, for more details).

12 We use the residuals of a regression of the openness index on country size and two dummies indicating whether the country is landlocked and oil exporter (Loayza et al. 2005).

13 There are two exceptions to this rule, although they represent extreme cases of limited empirical relevance: First, when poverty is extremely high (so nearly everyone is poor), a sufficiently large increase in inequality will push some individuals above the poverty line and thereby reduce poverty; second, when everyone is very rich (so poverty is close to zero), a sufficiently small increase in inequality will fail to push anyone below the poverty line, leaving poverty unchanged (see Marrero and Servén, 2018, for further elaboration and some numerical simulations illustrating this issue).

14 See Table 7 for the baseline case of headcount poverty with a US$ 2 poverty line. For alternative poverty lines and measures, the $${\mathrm{R}}^{2}$$ ranges from 0.303 for P2 (US$-1.25) to 0.582 for P0 (US$-4).

15 The distinction between uncorrelatedness and independence is important here. Our measure of poverty is constructed as a (exact) nonlinear function of log-income and the Gini. This implies that the data do not contain shocks to poverty independent from (as opposed to uncorrelated with) inequality and average income—indeed, the sample variation in poverty is not due to shocks correlated with income and inequality is necessarily due to shocks correlated with various nonlinear functions of these variables. Although we can estimate a linear equation such as (3), the interpretation of the resulting coefficient estimates requires some caution. For this reason, the discussion below focuses primarily on the empirical estimates of (1) and (2), and we view the estimation of (3) as a robustness check.

16 We initially constructed the instrument matrices using the second and higher lags of the variables (*s* > 1), as outlined in the text. However, the test of second-order serial correlation of the first-differenced residuals (the *m2*-test of Arellano and Bond 1991) rejected the null of no serial correlation in most specifications. Hence, we opted for lagging the instruments one more period, so that they are valid even in the presence of second (but no higher)-order serial correlation of the first-differenced residuals. To check this assumption, we report a test of third-order serial correlation of the first-differenced residuals.

17 Following López and Servén (2015), we drop Nigeria and Swaziland because of the poor quality of their GDP data. We found three big outliers for investment prices and six for inflation. These observations affect the Hansen tests of system GMM, bringing them closer to rejection in some cases, but have only minor incidence on the estimations.

18 Data for the infrastructure index included in M4 are available for only 88 countries under system GMM and 79 under the first-difference GMM specification. Using two lags as instruments to estimate this model would result in the number of instruments exceeding the cross section dimension of the data. Thus, we limit the number of instruments to just one lag.

19 The robustness exercises in section V follow the same pattern regarding cross-sectional dependence tests: The residuals of models M3 and M4 show no evidence of dependence, while in most cases, those of model M3 yield the opposite conclusion. Model M1 again yields mixed results.

20 The absence of cross-sectional dependence in models M3 and M4 (and, to a lesser extent, M1) may seem surprising given that short-term growth fluctuations typically display significant international comovement. However, our use of 5-year averages greatly mitigates the comovement usually found at annual (or higher) frequency. In addition, the inclusion of time dummies in our empirical specifications also helps soak up common factors affecting growth in multiple countries. Lastly, the presence of statistically significant policy variables in models M3 and M4 likely helps soak up any remaining cross-sectional correlation in these specifications, unlike in models M1 and M2.

21 The nonparametric part is approximated by a spline interpolation (Newson 2000), which yields similar results to the classical Epanechnikov-kernel-weighted local polynomial fit, but is recommended to approximate complex nonlinear shapes.

22 There are other institutional dimensions, such as the control of corruption, the military in power, the degree of international conflicts, or the Polity2 variable (from the Polity IV project). Including all these dimensions/variables simultaneously would introduce serious problems of collinearity in the estimated model.

23 The model in Marrero and Servén (2018) provides a theoretical foundation for these two simultaneous effects. In the model, the first term in (4) is associated with the direct impact of inequality on growth due to changes in the investment of the non-poor, whose sign is shown to be ambiguous—it depends on the degree of concavity of the production function. The second term represents the indirect impact of inequality on growth channeled through poverty, which is the focus of this section.

24 We obtain similar results when using ${\updelta }_{0}$ from (1).
